# β-CATENIN stabilizes HIF2 through lncRNA and inhibits intravenous immunoglobulin immunotherapy

**DOI:** 10.3389/fimmu.2023.1204907

**Published:** 2023-09-08

**Authors:** Chad Nakagawa, Manjunatha Kadlera Nagaraj, Juan Carlos Hernandez, Dinesh Babu Uthay Kumar, Vivek Shukla, Risa Machida, Jörg Schüttrumpf, Linda Sher, Patrizia Farci, Lopa Mishra, Stanley M. Tahara, Jing-Hsiung James Ou, Keigo Machida

**Affiliations:** ^1^ Department of Molecular Microbiology and Immunology, University of Southern California, Los Angeles, CA, United States; ^2^ University of Texas MD Anderson Cancer Center, Houston, TX, United States; ^3^ Biotest AG, Dreieich, Germany; ^4^ Department of Surgery, University of Southern California, Los Angeles, CA, United States; ^5^ Hepatic Pathogenesis Section, Laboratory of Infectious Diseases, National Institute of Allergy and Infectious Diseases, National Institutes of Health, Bethesda, MD, United States; ^6^ Southern California Research Center for Alcoholic Liver Disease and Pancreatic Disease (ALPD) and Cirrhosis, Los Angeles, CA, United States

**Keywords:** (HCC) hepatocellular carcinoma, (TIC) tumor-initiating cell, (HBV) hepatitis B virus, (DEN) diethylnitrosamine, (HBsAg) hepatitis B surface antigen, (FITC) fluorescein isothiocyanate, (HBIG) hepatitis B immunoglobulin

## Abstract

**Introduction:**

Tumor-initiating cells (TICs) are rare, stem-like, and highly malignant. Although intravenous hepatitis B and C immunoglobulins have been used for HBV and HCV neutralization in patients, their tumor-inhibitory effects have not yet been examined. Hepatitis B immunoglobulin (HBIG) therapy is employed to reduce hepatocellular carcinoma (HCC) recurrence in patients after living donor liver transplantations (LDLT).

**Hypothesis:**

We hypothesized that patient-derived intravenous immunoglobulin (IVIG) binding to HCC associated TICs will reduce self-renewal and cell viability driven by β-CATENIN-downstream pathways. β-CATENIN activity protected TICs from IVIG effects.

**Methods:**

The effects of HBIG and HCIG binding to TICs were evaluated for cell viability and self-renewal.

**Results:**

Inhibition of β-CATENIN pathway(s) augmented TIC susceptibility to HBIG- and HCIG-immunotherapy. HBV X protein (HBx) upregulates both β-CATENIN and NANOG expression. The co-expression of constitutively active β-CATENIN with NANOG promotes self-renewal ability and tumor-initiating ability of hepatoblasts. HBIG bound to HBV+ cells led to growth inhibition in a TIC subset that expressed hepatitis B surface antigen. The HBx protein transformed cells through β-CATENIN-inducible lncRNAs *EGLN3-AS1* and *lnc-β-CatM*. Co-expression of constitutively active β-CATENIN with NANOG promoted self-renewal ability of TICs through EGLN3 induction. β-CATENIN-induced lncRNAs stabilized HIF2 to maintain self-renewal of TICs. Targeting of *EGLN3-AS1* resulted in destabilization of EZH2-dependent β-CATENIN activity and synergized cell-killing of TICs by HBIG or HCIG immunotherapy.

**Discussion:**

Taken together, WNT and stemness pathways induced HIF2 of TICs via cooperating lncRNAs resulting in resistance to cancer immunotherapy. Therefore, therapeutic use of IVIG may suppress tumor recurrence through inhibition of TICs.

## Introduction

Hepatocellular carcinoma (HCC) is the fourth most common cancer, accounting for 80% of liver cancer cases, and is the third deadliest form of cancer (1–3). Clinical studies indicate that 40% of HCCs have clonality and are considered to originate from progenitor/stem cells (4, 5). In addition to virus protein-mediated carcinogenic mechanisms, the integration of viral genes promotes viral carcinogenesis, especially in the case of hepatitis B virus. The tumor-initiating cells (TICs) are rare, stem cell-like cells that play a central role in tumorigenesis, malignant progression, and resistance to chemotherapy (6, 7). This resistance to chemotherapy is potentially due to the oncogenic signaling pathways activated in these cells.

The Milan criteria restrict liver transplantation for an adult with HCC who has a single tumor diameter of less than 5 cm or not more than three tumor foci, each not exceeding 3 cm with no angioinvasion nor extrahepatic involvement. Liver transplantation is effective for small, unresectable HCC patients with cirrhosis (8). High-dose hepatitis B immunoglobulin (HBIG) therapy is commonly employed for HCC patients exhibiting HBV-DNA/hepatitis B *e* antigen (+) as an adjunct following living donor liver transplantation (LDLT) (9). This HBIG therapy reduces HCC recurrence in HBV-DNA/HBeAg(+) LDLT patients. HCC recurrence and overall survival of those who do not meet the Milan criteria are unaltered independent of the HBIG dose (10). Furthermore, the presence of TICs and/or invasive tumor characteristics inhibit HBIG efficacy for liver transplantation for HCC patients with cirrhosis. An understanding of the pathways involved in generating and maintaining HCC TICs will provide insight into effective treatment strategies.

As a contributor to more than 50% of HCC cases worldwide (11), hepatitis B virus (HBV)-mediated signaling underlies HCC tumorigenesis. The HBV-encoded HBx protein promotes HCC development through viral replication and stemness (12). To assist an examination of the tumorigenic effects of HBx, HBV transgenic mice with or without HBx were developed (13–16). When these mice were treated with the genotoxic agent diethylnitrosamine (DEN), HBx-positive mice showed enhanced tumorigenesis compared to the HBx negative counterparts, with tumor incidences of 91% in HBx-positive vs. 63% in HBx-negative mice (13–15). This transgenic mouse model has proven to be a translational tool for the study of HBx-mediated development of HCC.

The interplay between HBx, β-CATENIN, and other signaling molecules to promote malignancy and TIC formation has not been fully elucidated. β-CATENIN/CBP signaling promotes HCC tumor occurrences through β-CATENIN/CBP-target gene expression, including MYC, CYCLIN D1 and OCT4. It is important to determine *in vivo* how HBV-mediated beta-CATENIN signaling promotes stemness and tumor recurrence/therapy resistance. 

The roles of β-CATENIN in liver disease progression have been established *in vitro* and *in vivo*. Intravenous immunoglobulin (IVIG) immunotherapy is an established practice. Cancer resistance to therapy is seen in the clinic and one of several contributory driver pathways is β-CATENIN signaling. The mechanism(s) as to how beta-CATENIN signaling promotes immunotherapy resistance and IVIG treatment resistance mechanisms are not fully understood. The pluripotency transcription factor NANOG promotes stemness and reprograms metabolism which promote therapy resistance and self-renewal (17). Notably, NANOG is used as one of Thompson’s iPS reprograming factors besides LIN28, SOX2 and OCT4 (18).

HBIG treatment after liver transplantation suppresses HCC recurrence and extends the graft recipient’s survival. The molecular mechanisms regarding HBIG treatments for suppressing HCC recurrence, however, have not been fully elucidated. We hypothesized that HBIG and HCIG bind TICs and CTCs to remove these residual sources of tumor recurrence. Activation of β-CATENIN signaling antagonizes HBIG/HCIG immunotherapy to allow HCC recurrence and metastasis. The aim of these studies was to characterize the HBx-mediated signaling through β-CATENIN that promotes tumor malignancy, IVIG therapy resistance, and to evaluate the effect of HBIG and HCIG treatments on this pathway as well as on overall HCC TIC renewal and viability.

## Results

### β-CATENIN and NANOG are overexpressed in HBV transgenic mice

**Figure 1:**
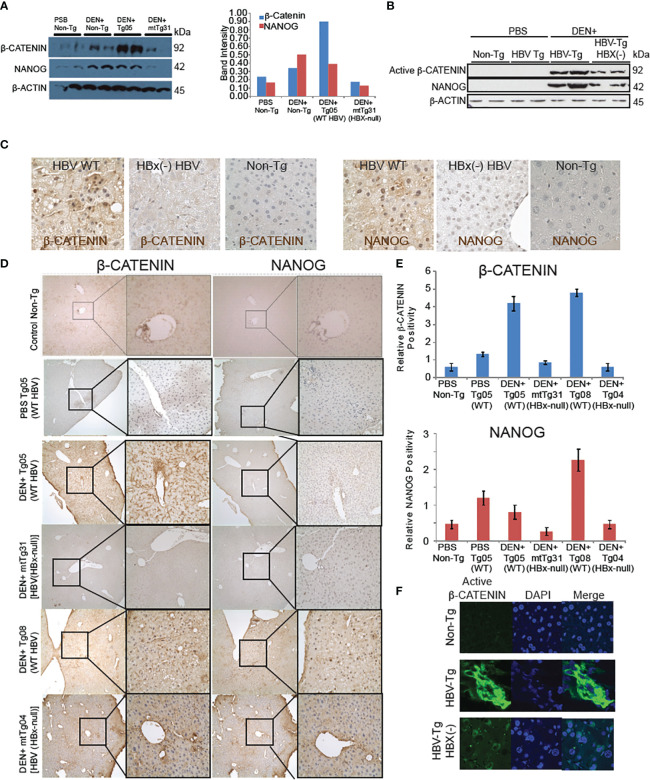
β-CATENIN and NANOG are induced in livers of HBV transgenic mice dependent on the HBx gene. **(A)** Immunoblot analysis of β-CATENIN and NANOG levels, standardized to β-ACTIN levels, in transgenic mouse tissue. **(B)** HBx regulation of β-CATENIN and NANOG levels. The Tg05 mouse sample has a full-length 3.2-kb HBV genome and the mtTg31 mouse sample has two point-mutations in the HBx gene to prevent its expression. The β-CATENIN molecular weight is 94kDa, the NANOG molecular weight is 34kDa, and β-actin is 42kDa. Non-phospho (active) β-CATENIN (Ser33/37/Thr41) reacts with active form of β-CATENIN. Band intensities in the graph on the left were standardized to the β-actin levels. **(C)** Immunohistochemistry staining of β-CATENIN and NANOG in mouse liver tissues using 200× (left) and 400× (right) magnification. Positive cells were counted in 10 high-power fields (400×), and the average was taken (bottom). Immunofluorescence **(B)** and immunohistochemical analysis **(C)** confirmed active β-CATENIN and NANOG in mouse tissue sections from HBV WT Tg mice. **(D)** Comparative analysis between β-CATENIN and NANOG expression in HBV transgenic mouse models with or without DEN. DEN^+^ Tg05 and DEN^+^ Tg08 both show significantly higher expression of β-CATENIN and Tg05 has a significantly higher expression of NANOG. **(E)** In both samples, both β-CATENIN and NANOG are localized in the nucleus, unlike mtTg04 and mtTg31. **(F)** Elevated levels of β-CATENIN in transgenic mouse liver tissue. Immunofluorescent staining of β-CATENIN (19) using 400x magnification. Nuclear fluorescence was counted in 10 high-power fields and the average was taken. Non-phospho (Active) β-CATENIN (Ser33/37/Thr41) detects active form of β-CATENIN. Refer to **Supplementary Figure 2**.

An HBV transgenic mouse model was used to evaluate pathways that may work together with the WNT/β-CATENIN pathway to promote HCC tumor occurrence. The DEN^+^ mtTg31 mouse line harbors an HBV genome that is missing HBx. These HBV Tg mice were injected with the genotoxic carcinogen diethylnitrosamine (DEN) and maintained 10 months after DEN injection and euthanized. These mice exhibited NANOG and β-CATENIN levels that were both reduced compared to the DEN^+^ Tg05 line which expressed an intact HBV genome (**Figures 1A, B**, **Supplementary Table 8**). This indicated that HBx was required for the increased expression of *β-CATENIN* mRNA in the transgenic mouse liver samples.

Wild-type HBV transgenic mice showed significantly higher levels of β-CATENIN in liver samples compared to mutated HBx HBV mice, and the former showed a larger proportion of β-CATENIN localizing to the nucleus (*P* < 0.05) (**Figures 1C, D**). In transgenic mice treated with DEN, NANOG levels were significantly higher in the Tg05 and Tg08 samples compared to the mtTg31 and mtTg04 samples. For the samples showing nuclear localization (DEN^+^ Tg05 and DEN^+^ Tg08), the β-CATENIN and NANOG staining appeared to overlap. The DEN^+^ Tg05 sample β-CATENIN staining was positive near the portal triad, whereas in the DEN^+^ Tg08 sample β-CATENIN staining was positive near fatty liver and dysplastic tissue (**Supplementary Figure 1**). To further confirm the overlap of β-CATENIN and NANOG in the nuclei, we double-stained the tissue samples using fluorescent antibodies and only saw nuclei positive for both proteins in DEN^+^ Tg05 and DEN^+^ Tg08 (**Figures 1D–F**).

### shRNA silencing-low-throughput screenings validated that ARID1A silencing promotes stemness, but silencing of EZH2, SUZ12 and EED reduces stemness

**Figure 2:**
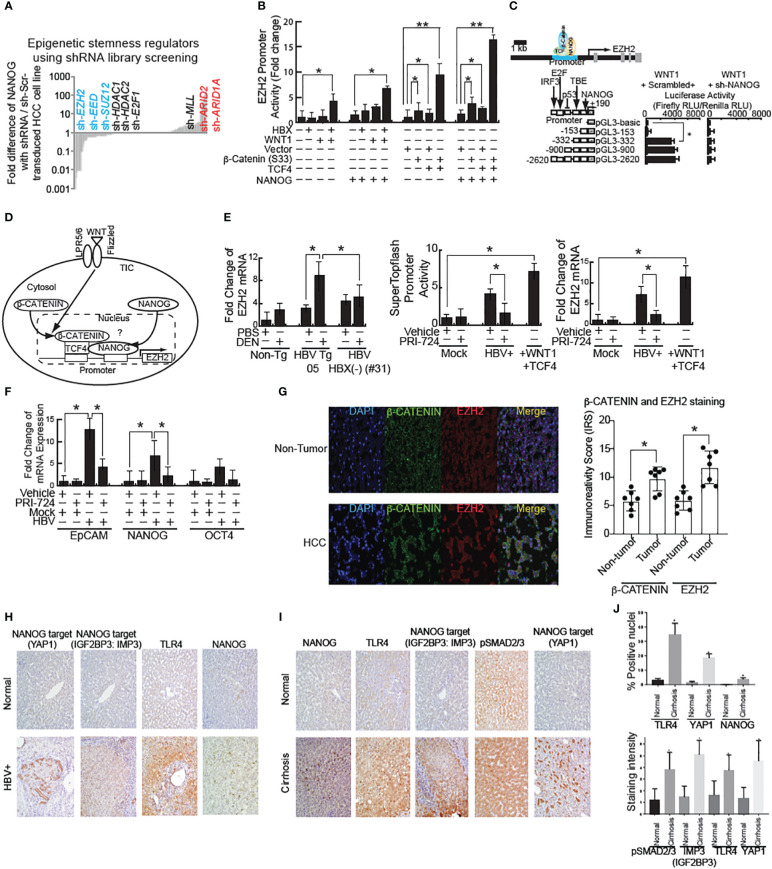
shRNA screening of stemness-inducers identified EZH2 that is transactivated by WNT and NANOG. **(A)** High-throughput shRNA screening of epigenetic regulators for stemness identified that EZH2 silencing reduced stemness genes. **(B)** EZH2 promoter activity in HBX- or active β-CATENIN–transfected cells. β-CATENIN (S33) is wild type β-CATENIN-expression vector without constitutive activation mutation in exon 3 stabilization domain. TCF4: DNA-binding domain for β-CATENIN-complex for transactivation of target genes. *: Asterisk denotes statistical significance (P < 0.05). **: denotes statistical significance (P < 0.01). **(C)** EZH2 promoter luciferase reporter assay. **(D)** Schematic of WNT–mediated EZH2 transactivation through β-CATENIN/TFC4 and NANOG. WNT signal activation stabilizes β-CATENIN to transactivate target gene, including NANOG. Bioinformatics analyses identified that β-CATENIN and NANOG both bind to the EZH2 promoter regions to transactivate EZH2 mRNA. **(E)** Left and Right: WNT-inducible gene induction in HBV-infected cells. β-CATENIN-CBP inhibitor PRI-724 inhibits HBV-mediated WNT activation. Middle: SuperTopflash activity in HBV-infected cells. **(F)** HBV-Tg mouse showed WNT pathway activation by mRNA level. **(G)** (Left) Immunostaining for EZH2 and β-CATENIN in human HCC tissues associated with HBV infection. β-CATENIN normally localizes around membranes. In HCC specimens, immunoreactivity scores were significantly increased. (Right) Quantitation of EZH2 staining intensity in human HCC tissues. The Y-axis units in the graph depict immune reactivity score (IRS). **(H)** Increased expression of TLR4, NANOG, and its downstream genes (YAP1 and IGF2BP3) in human cirrhotic livers samples. **(I)** Significant increase in nuclear expression of NANOG, TLR4 and YAP1 in cirrhotic livers samples in comparison to normal liver (*P* < 0.05). **(J)** Significant increase in pSMAD2/3, IMP3 (IGF2BP3), TLR4 and YAP1 cytosolic expression was observed (*P* < 0.05) in cirrhotic livers in comparison to normal liver.

To determine which epigenetic genes promote stemness gene expression, including NANOG, *in vitro* shRNA-silencing screening analysis was performed in HCC cell lines and TICs isolated from HCC patients. Potential epigenetic regulators that promote tumorigenesis were further assayed by shRNA silencing-based, low-throughput screenings (72 total candidate screenings); this confirmed that Polycomb repressive complex 2 (PRC2) complexes were significant NANOG regulators (**Figure 2A**). Notable among these genes were *Ezh2* (20)*, Eed* and *Suz12*. An enzyme subunit of PRC2 protein complexes is EZH2 that methylates histone 3 lysine 27 (H3K27me3), which suppresses gene expression by facilitating chromatin condensation and recruitment of several demethylases to remove active histone marks (H3K4me3). We validated the predicted synthetic lethality targets of these PRC2 components, as previously identified by GeCKO-lentivirus library screening against a mutant *arid1A* background. We hypothesize that the loss of ARID1A activity leads to the elevated PRC2 activity resulting in inactivation of polycomb target genes (including OXPHOS genes) through H3K27-trimethylation. The latter chromatin modification would lead to the hyperactivation of the stem cell gene programs, including NANOG induction. H3K27 trimethylation represses PRC2 target genes (21, 22) although another possibility is that H3K27 trimethylation activates other gene loci which in turn inactivate OXPHOS.

We next examined the relationship between NANOG and PRC2 on the regulation of OXPHOS genes in TICs. ARID1A and ARID2 genes encode a member of the SWI/SNF family, whose members have helicase and ATPase activities and alter chromatin structure to regulate transcription of specific genes (23). The encoded ARID protein is part of the large ATP-dependent chromatin remodeling complex SWI/SNF, which is required for transcriptional activation of genes normally repressed by chromatin. ARID protein of the SWI/SNF complex possesses two conserved domains for DNA-binding to AT-rich DNA sequences.

An shRNA library screening identified ARID1A and ARID2 among the most likely negative epigenetic regulators of NANOG (**Figure 2B**). Although other possible regulators were identified, such as P53 and TCF3 protein TCF (T-Cell Specific, HMG-Box) binding within the NANOG promoter regions (24). As a mimic for previously described endotoxin effects, RT-qPCR analysis of LPS-treated TICs resulted in higher NANOG mRNA expression compared to primary hepatocytes. Knockdown of ARID1A further amplified the LPS-induced NANOG expression in TICs (**Figure 2C**). These results showed that loss of ARID1A function inactivated SWI/SNF complex leading to augmented NANOG expression in TICs, implying that SWI/SNF silenced NANOG. Thus, the role of endotoxin *in vivo* is expected to increase NANOG expression and ARID1A activity is needed to augment SWI/SNF. Based on these findings, we hypothesized that cancer-promoting mutations (i.e., ARID1A) increase PRC2 complex activity (including expression of the EZH2 component) and induce an HCV/alcohol-mediated stem cell program (slow growing cells), leading to TIC-initiated HCC development (**Figure 2C**).

### Cooperation of β-CATENIN and NANOG signaling to transactive EZH2

Using the Oncomine program we searched for keywords including metastasis, liver cancer, hepatocellular carcinoma, and hepatitis B virus. We compared the ranked gene lists of many different studies against the gene list compiled from our lab’s previous NANOG ChIP-seq data (17). One of the more relevant top ranked relevant genes was *Ezh2*, found in the Ye et al. Oncomine data set. These data identified HBV-positive metastatic HCCs by use of gene expression profiling and supervised machine learning (25) in order to choose relevant pathways.

We used Biobase to examine the *Ezh2* promoter region for the presence of β-CATENIN or NANOG binding sites and were able to identify potential binding sites for both factors. EZH2-mediated, epigenetic silencing of Wnt antagonists constitutively activates WNT/β-CATENIN signaling, leading to HCC proliferation through de-repression of the growth-suppressive AXIN2, NKD1, PPP2R2B, PRICKLE1, and SFRP5 (26). To test if HBV infection activates WNT signaling, the HBV cell line was transfected with the EZH2 reporter, followed by treatment in the presence or absence of β-CATENIN-CBP interaction inhibitor PRI-724. The WNT ligand stimulation induced EZH2 promoter activities (**Figure 2D**). HBV stimulated EZH2 promoter reporter activity, which was inhibited by PRI-724 WNT/CBP antagonist treatment (**Figures 2D, E**). This indicated that HBV induced EZH2 (**Figure 2F**). The PRI-724 treatment also efficiently blocked the expression of endogenous EZH2 in normal liver (**Figure 2G**, right).

HBX-transfected cells express the bipotential marker EpCAM as well as the stemness markers NANOG and SOX2 at levels two- to six-fold higher than control cells. To test if PRI-724 treatment inhibited additional WNT-inducible target genes, HBV-infected cell lines treated in the presence or absence of PRI-724 were examined for effects on stemness genes *NANOG*, *EpCAM* and *OCT4* (**Figure 2F**). The expression levels for EpCAM, NANOG and OCT4 were reduced by PRI-724 treatment (**Figure 2F**).

HBV X protein (HBX) is known to activate the WNT pathway (27). We observed β-CATENIN levels were reduced in some of the HBX(-) HBV Tg mice (**Figure 1B**, Right, last two lanes). To test if the WNT pathway is activated in an HBV-mediated liver disease model, mRNA, protein and immunostaining of liver tissue sections was performed. HBV-Tg05 mouse liver has elevated EZH2 expression (**Figure 2G**) and active β-catenin (**Figure 2G**). Colocalization of β-catenin and EZH2 was observed in HCC tissues, but less frequently in adjacent, non-cancerous tissues (**Figure 2G**). These results indicated that HBV-induced fibrosis/HCC was associated with activation of the WNT pathway in the HBV-transfected human cell line and our HCC mouse model.

### Pluripotent stem cell transcription factor NANOG is elevated in liver diseases associated with HBV

Previous studies have shown a significant increase in TLR4 and two NANOG-target genes (28), including IMP3 (IGF2BP3) (**Figures 2H–J**, **Supplementary Figures 5–8**) and YAP1 where increased cytosolic expression was observed (*P*<0.05) in liver tumors compared to normal livers (**Figures 2H–J**, **Supplementary Figures 5–8**). By contrast pSMAD2/3 was downregulated in liver tumors compared to normal livers (**Supplementary Figures 5**), indicating that NANOG may transactivate or induce phosphorylation of these target genes. Activation of TLR4 (29) was validated in these HBV-associated liver diseases. Our data further showed a significant increase in nuclear expression of TLR4, NANOG and NANOG-downstream gene activation in liver tumor samples in comparison to normal liver (shown in **Supplementary Figures 5–8**; *P*<0.05).

### β-CATENIN and EZH2 differentially regulate downstream target genes through lncRNA-β-CatM

The transcription factor TCF (T-Cell Specific, HMG-Box) gene encodes a member of the T-cell factor/lymphoid enhancer-binding factor family of high mobility group (HMG) box transcriptional activators. This gene is expressed predominantly in T-cells and plays a critical role in natural killer cell and innate lymphoid cell development. The encoded protein forms a complex with β-CATENIN and activates transcription through a WNT/β-CATENIN signaling pathway.

**Figure 3:**
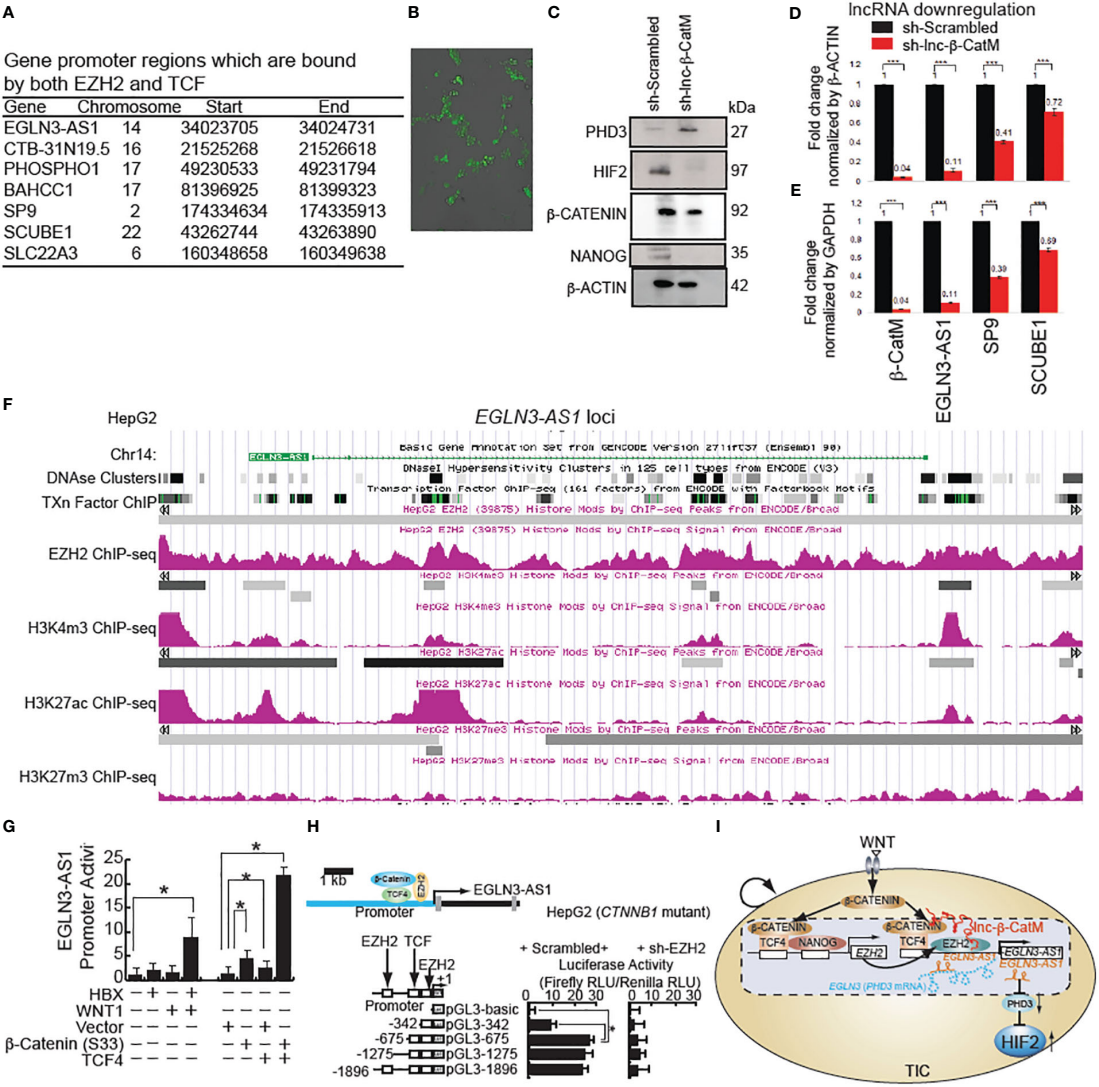
β-CATENIN transactivates human lncRNA EGLN3-AS1(antisense sequence of HIF-2 inhibitor protein-coding gene: EGLN3) in the presence of lncRNA β-CatM. **(A)** Genes that are bound by both human EZH2 and TCF with locations of binding. **(B)** GFP expression in Huh7 cells after incubation with lentivirus-expressing shRNA targeting EGLN3-AS1-GFP and after puromycin selection. **(C)** Immunoblot analysis showing the increase in expression level of PHD3 after knockdown of *lnc-β-CatM*. **(D, E)** RT-qPCR results showing the expression levels of the indicated genes after knockdown of *lnc-β-CatM* in comparison to housekeeping gene β-Actin in **(D)** and glyceraldehyde 3-phosphate dehydrogenase (GAPDH) in **(E)**. **(F)** ***: denotes statistical significance (P < 0.001). ChIP-seq data for EZH2, positive chromatin marks (H3K4me3 and H3K27ac) on *EGLN3-AS1* gene loci (EGLN3: PHD3-coding gene) from HepG2 cell line. **(G)** LncRNA *EGLN3-AS1* promoter activity in Huh7 cells (wild type *CTNNB1*: *β-CATENIN*-coding gene). **(H)** EZH2 silencing reduces *EGLN3-AS1* promoter activity by use of luciferase reporter assay in HepG2 cells (constitutively active mutant of β-catenin). **(I)** Hypothetical model that β-CATENIN-NANOG induces EZH2 that transactivates lncRNA *EGLN3-AS1* (antisense lncRNA for PHD3-coding gene *EGLN3*) with β-CATENIN to silence the PHD3 gene and in turn to stabilize HIF2 proteins in TICs.

Next, we analyzed the ChIP-seq data for EZH2 and TCF, which formed a complex with β-CATENIN, within regions where both the proteins colocalize (30). The BaseSpace correlation engine software was used to determine the expression levels of these genes in HCC for comparison to adjacent normal tissues (**Figure 3A**). Seven regions in the genome were identified from the ChIP-seq data analysis for both EZH2 and β-CATENIN binding (**Figure 3A**). GFP reporter expression in the Huh7 cells was examined after incubation with lentivirus and after puromycin selection to monitor transduction efficiency to examine the percentage of cells transduced with lentiviral GFP constructs that encode shRNA genes of interest. The cells were visualized by fluorescent microscopy after puromycin selection to check for the production of GFP (**Figure 3B**). As β-CATENIN and NANOG transactivate EZH2 and oncogenesis, PRC2 complex-binding lncRNAs were also screened. Among them, lnc-β-CatM is the top candidate for EZH2-bound lncRNAs that are upregulated in HCC patients (31). lncRNA β-CatM binds EZH2 to promote oncogenesis, thereby promoting β-CATENIN methylation (31). lnc-β-CatM is required for self-renewal of liver TICs and tumor propagation in mice (31). Methylation suppresses the ubiquitination of β-CATENIN and promotes its stability, leading to activation of WNT-β-CATENIN signal (31).

Prolyl hydroxylase domain-containing protein 2 (PHD2) catalyzes the post-translational formation of 4-hydroxyproline in HIF-1α proteins and leads to destabilization of HIF-1α whereas PHD3 mainly destabilizes the HIF-2α isoform (32–34). HIF is a transcriptional complex that plays a central role in mammalian oxygen homeostasis. PHD2 senses cellular oxygen, and under normoxia, modification by prolyl hydroxylation promotes proteasomal destruction of HIF subunits via the von Hippel-Lindau ubiquitylation complex (35). Therefore, we postulate that inhibition of antisense gene (EGLN3-AS1) expression for its effect on PDH3 leads to stabilization of HIF-2α in hypoxic microenvironment. Immunoblot analysis showed an increase in expression of PHD3 after knockdown of lnc-β-CatM (**Figure 3C**). The following genes were upregulated in liver cancer: CTB-31N19.5 (methyl transferase like 9), BAHCC1 (Bromo Adjacent Homology domain and Coiled-Coil Containing 1), SCUBE1 (Signal Peptide, CUB Domain and EGF Like Domain Containing 1). By contrast, PHOSPHO1, SP9, SLC22A3 (Solute Carrier Family 22 Member 3) were downregulated. The expression level of EGLN3-AS1 was not found using BaseSpace software.

### LncRNA lnc-β-CatM knockdown reduces β-*Catenin* signaling to reduce lncRNA *EGLN3-AS1*

Huh7 cells were infected with the lentivirus expressing sh-lnc-β-CatM. Knockdown of lnc-β-CatM reduced the expression level of the antisense RNA EGLN3-AS1, which binds EGLN-3 mRNA to inhibit protein expression (31). Accordingly, the reduction in EGLN3-AS1 (this gene encodes antisense RNA which is complementary to PHD3 mRNAs) increases the expression levels of the protein coding gene EGLN3 (**Figure 3C**). This result was consistent with the chromosomal location of EGLN3-AS1 and EGLN3 where reduction of the former would lead to a proportional increase in EGLN3.

After knocking down *lnc-β-CatM*, q-PCR was done to measure the efficiency of knockdown; for comparison, cells infected with lentivirus expressing scrambled shRNA (Scr) were used as control. The knockdown efficiency of the sh-RNA was high, with 96% reduction in *lnc-β-CatM* (**Figure 3D**). The expression levels of the seven genes that were bound by both EZH2 and β-CATENIN were examined as a consequence of this knockdown. The knockdown of *lnc-β-CatM* resulted in reduction of the expression levels of *EGLN3-AS1,* SP9, and SCUBE-1. The other genes did not show any statistical differences in expression compared to the control shRNA (**Figure 3E**). The *EGLN3-AS1* locus showed enrichment of both H3K27ac and EZH2 ChIP-seq peaks, indicating that active chromatin marks were present in *EGLN3-AS1* regulatory regions (**Figure 3F**). This indicated that even if repressive complexes were present in the *EGLN3-AS1* locus, active chromatin marks showed enrichment as well.

Among these target genes, *EGLN3-AS1* stands out because EGLN3 (PHD3) is a prolyl hydroxylase activity which downregulates HIFs by increasing protein turnover and is a suppressor of HIF-2α. In cancer cells, the expression of HIF target genes is activated under hypoxic conditions (36). Multiple epigenetic modifications in the upstream region of *EGLN3-AS1* show that the gene is activated in cancer. We hypothesized that β-CATENIN and EZH2 transactivate *EGLN3-AS1* to downregulate EGLN3 in HepG2 cells (resulting in constitutively active β-CATENIN). ChIP-seq data for H3K4me3 and H3K27ac from the HepG2 cell line showed peaks in the upstream region of *EGLN3-AS1* (**Figure 3G**). H3K4me3 and H3K27ac marks are mostly enriched at active promoter regions near transcription start sites and positively correlate with transcription. The DNase hypersensitivity cluster regions showed that accessibility of this promoter region also correlated with the activating histone marks. WNT stimulation transactivated lncRNA *EGLN3-AS1* promoter activity in Huh7 cells (wild type β-CATENIN) (**Figure 3H**). By contrast, EZH2 silencing reduced *EGLN3-AS1* promoter activity shown by luciferase reporter assay in HepG2 cells (constitutively active mutant of β-CATENIN) (**Figure 3I**). These results indicated that β-CATENIN and EZH2 transactivate EGLN4-AS1 to downregulate PHD3, leading to induction of HIF2α protein.

### shRNA silencing-based, low-throughput screenings validate that ARID1A silencing promotes stemness, but silencing *EZH2, SUZ12* and *EED* reduces stemness

**Figure 4:**
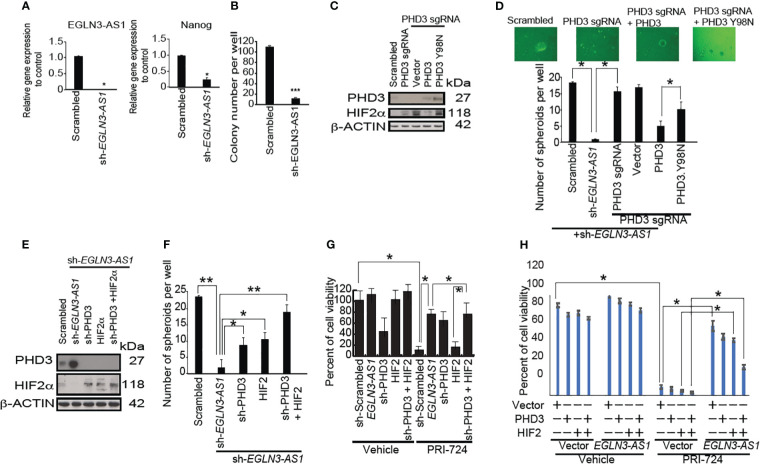
LncRNA *EGLN3-AS1* suppresses EGLN3 to stabilize HIF2α that is inhibited by β-CATENIN inhibition. **(A)** Silencing of *EGLN3-AS1* significantly reduced expression (n=3, *p< 0.05). **(B)** Silencing of *EGLN3-AS1* significantly reduced the number of colonies appearing in soft agar assay (n=4, ***p< 0.001). **(C)** CRISPR/Cas9-mediated knockout of PHD3 and restoration of PHD3 wild type and mutant (PHD3.Y98N: phosphatase domain mutant) were confirmed by immunoblots. **(D)** PHD3 inhibits *EGLN3-AS1* mediated self-renewal of TICs via phosphatase domain. Silencing of *EGLN3-AS1* significantly reduced the number of spheroids (n=3, *p< 0.05). **(E)** Silencing PHD3 in TICs with shRNA targeting *EGLN3-AS1* was confirmed by immunoblot analyses. **(F)** Silencing PHD3 restored self-renewal ability of TICs transduced with shRNA targeting *EGLN3-AS1*. *: P < 0.05; **: P < 0.01. **(G)** Silencing PHD3 rescued β-CATENIN-CBP inhibitor PRI-724-mediated killing of TICs transduced with shRNA against *EGLN3-AS1*. **(H)** Overexpression of PHD3 in TICs with *EGLN3-AS1* overexpression restored killing activity of TICs in response to β-CATENIN-CBP inhibitor PRI-724 treatment.

An *in vitro* analysis was performed in HCC cell lines and TICs isolated from HCC patients. Potential epigenetic regulators that promote tumorigenesis were further screened by shRNA silencing-based, low-throughput screenings (72 total candidate screenings), notable among these genes analyzed were *EZH2* (20)*, EED* and *SUZ12*. This analysis confirmed that PRC2 complexes were significant NANOG regulators (**Figure 4A**). We validated predicted synthetic lethality targets of these PRC2 components, as previously identified by GeCKO-lentivirus library screening against a mutant ARID1A background. Consequently, we hypothesized that the loss of ARID1A activity leads to elevated PRC2 activity resulting in inactivation of polycomb target genes (including OXPHOS genes) through H3K27-trimethylation. The latter chromatin modification would expectedly lead to the hyperactivation of the stem cell gene programs, including NANOG induction. H3K27 trimethylation represses PRC2 target genes (21, 22) although another possibility is that H3K27 trimethylation activates other gene loci which inactivate OXPHOS genes. We next examined the relationship between NANOG and PRC2 on the regulation of OXPHOS genes in TICs. For this purpose, an shRNA library screening identified ARID1A and ARID2 among the most likely negative, epigenetic regulators of NANOG (**Figure 4A**). This screening identified other possible regulators, such as P53 and TCF3 proteins binding within the NANOG promoter region (24).

### β-CATENIN inhibitor combination treatment targets the TIC population via suppression of *EGLN3-AS1*

To determine if *EGLN3-AS1* promoted self-renewal of TICs, we knocked down *EGLN3-AS1*, the RNA precursor of EGLN3-AS1 in TICs. Silencing EGLN3-AS1 reduced *NANOG* expression and decreased expansion of TICs (**Figure 4B**). Furthermore, when we subjected *EGLN3-AS1* silenced cells to tumor colony formation assays, the colony numbers were significantly reduced (**Figure 4B**). These results indicated that down-regulation of *EGLN3-AS1* was important for suppressing TIC self-renewal.

We performed CRISPR/Cas9-mediated knockout of endogenous PHD3 and replacement by PHD3 wild type and a mutant (PHD3.Y98N: phosphatase domain mutant) which were confirmed by immunoblots (**Figure 4C**). PHD3 restoration rescued sh-*EGLN3-AS1*-mediated inhibition of self-renewal of TICs via its phosphatase domain (**Figure 4D**). Silencing of PHD3 and HIF2 in TICs with shRNA targeting *EGLN3-AS1* was confirmed by immunoblot analyses (**Figure 4C**). Silencing EGLN3-AS1 induced PHD3 expression (**Figure 4E**). Silencing PHD3 or overexpression of HIF2alpha in TICs with shRNA targeting EGLN3-AS1 was confirmed by Western blot analyses (**Figure 4E**). The shRNA-mediated knockdown of *EGLN3-AS1* led to increased PHD3-protein levels that was abrogated by sh-PHD3 (**Figure 4F**). Silencing PHD3 restored self-renewal ability of TICs transduced with shRNA targeting *EGLN3-AS1* (**Figure 4G**). Silencing of PHD3 rescued β-CATENIN-CBP inhibitor-mediated killing of transduced TICs (**Figure 4G**, right). Vice versa, overexpression of *EGLN3-AS1* created the resistant phenotype of TICs. Overexpression of sh-PHD3 inhibited the killing effect induced by β-CATENIN-CBP inhibitor (PRI724) treatment (**Figure 4G**). Restoration of HIF2α and/or sh-PHD3 expression reversed β-CATENIN-CBP inhibitor (PRI724)-mediated killing of TICs transduced with the *EGLN3-AS1* gene (**Figure 4G**). Thus, these results demonstrated that the drug combination treatment induced TIC growth arrest and apoptosis through the PHD3 axis. To determine mechanism of action of PRC-724, EGLN3-AS1 resucue experiments were performed. EGLN3-AS1-medaited rescue effects were reversed by HIF2alpha overexpression. Overexpression of PHD3 in TICs with EGLN3-AS1 overexpression restored viability of TICs in response to PRC-724 treatments (**Figure 4H**).

### β-CATENIN, NANOG-target gene and β-CATENIN downstream gene (BIRC5) are elevated in human HCC tissues

Based on established relationships between β-CATENIN and NANOG-downstream gene YAP1 in upregulating BIRC5 (37, 38), we evaluated relationships between BIRC5, β-CATENIN, and NANOG expression levels with poor HCC prognosis or malignant transformation of HCC development. This *in silico* analysis identified a significant relationship between BIRC5 levels and liver cancer precursors (cirrhosis and fibrosis) compared to HCC (**Supplementary Figure 3**). To help physicians to decide which treatment is the more effective for HCC patients, the Barcelona Clinic Liver Cancer (BCLC) staging system was established which considers the number and sizes of tumors in livers and the general patient health and fitness (performance status). Higher BIRC5 levels were correlated with higher BCLC stage, tumor grade and with higher observed BIRC5 levels in metastatic sites in patients who died after 3 years of disease. This indicated that β-CATENIN-downstream genes are induced in late-stage cancers. Therefore, we postulated that β-CATENIN and pluripotency transcription factors may synergistically transactivate unique genes in TICs that are notably enriched in late-stage cancers.

**Figure 5:**
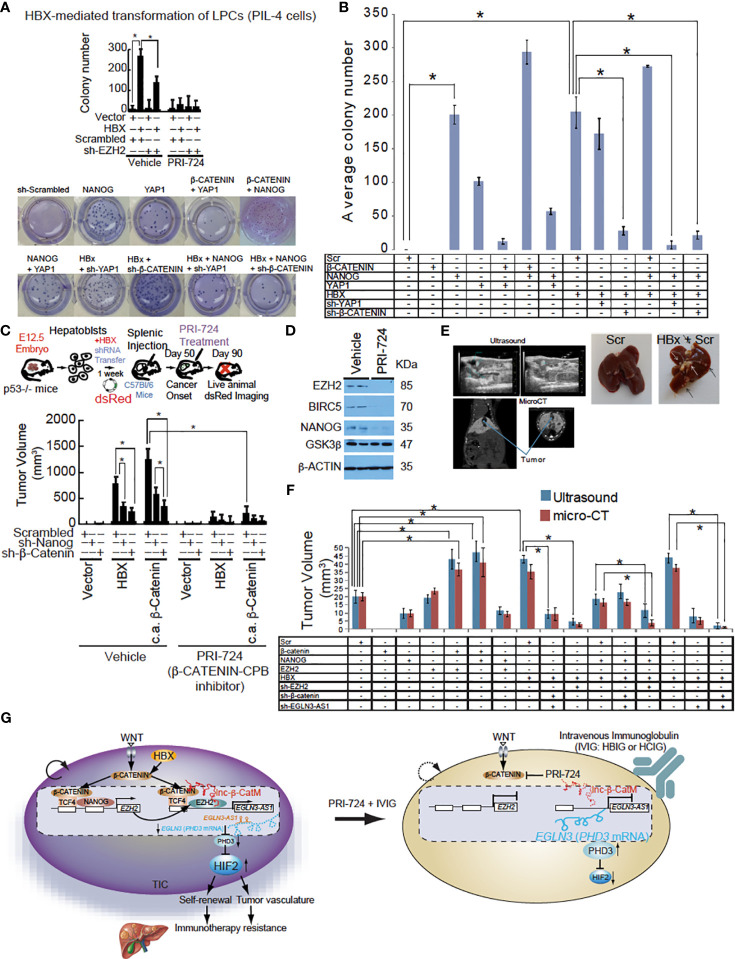
*EGLN3-AS1* or β-CATENIN targeting reduced HBX-mediated tumor growth. **(A)** Silencing of *NANOG* and *β-CATENIN* in hepatoblasts promotes growth inhibition of intrahepatic tumors achieved by β-CATENIN-CBP complex inhibitor PRI-724 in immune-competent C57BL/6 mice as monitored by dsRed imaging. Bottom: Colony formation assay of mouse hepatoblasts transfected with a combination of *NANOG, β-CATENIN*, Yap1, HBx, Scrambled shRNA, sh-β-CATENIN, or sh-YAP1. Active β-CATENIN and HBX transform liver progenitor cells in a manner dependent on NANOG and β-CATENIN. Silencing of *NANOG* sensitizes TICs to PRI-724-induced cell death. **(B)** Average number of colonies in three different wells shown. Colony formation assay was carried out using C57Bl/6 hepatoblasts and lentiviral or retroviral constructs were used to stably transfect each of the groups. β-CATENIN + NANOG showed the highest number of colonies and was significantly more than NANOG alone. This could potentially be due to NANOG being able to recruit CBP/p300 to β-CATENIN. *Denotes that β-CATENIN + NANOG had a significantly higher average number of colonies compared to any other group (*P* value < 0.05, two-tailed, unpaired, Student *t* test). **(C)** The tumor volume measurement performed at day 90 after intrahepatic transplantation, shows a maximal growth-retardation achieved by the combination of the PRC-724 and *Nanog/β-Catenin* silencing. Intrahepatic tumor growth by TICs from HCC patients in C57Bl/6 mice was significantly suppressed by PRC-724 treatment only in the presence of *Nanog* and *β-Catenin* silencing. Hepatoblasts transfected with HBx showed elevated tumor growth. Transduction of β-CATENIN, NANOG, HBX transforms hepatoblasts injected intrahepatically into C57Bl/6 mice. Top: HBX-mediated NANOG transactivation transforms hepatoblasts via activation of β-CATENIN. Intrahepatic tumor growth by TICs from HCC patients in C57Bl/6 mice was significantly suppressed by PRI-724 treatment only in the presence of NANOG and β-CATENIN silencing. Bottom: Tumor volumes at day 90 after intrahepatic transplantation. **(D)** The immunoblot analysis of cells treated with PRI-724 or vehicle at day 90 after intrahepatic transplantation. **(E)** Top: Representative images of ultrasound and micro-CT of mouse livers. Bottom: Liver images from euthanized mice. **(F)** Tumor measurements from ultrasound and micro-CT provided by the animal imaging core at the University of Southern California radiology department. **(G)** Hypothetical model that WNT-mediated β-CATENIN and NANOG transactivate EZH2 that cooperatively induces lncRNA *EGLN3-AS1* (antisense oligonucleotides that is expressed in cancerous tissues with β-CATENIN that destabilizes PHD3 and in turn stabilizes HIF2α proteins, leading to liver oncogenesis and immunotherapy resistance. RPI724 and HBIG combination treatment significantly reduced tumor growth and completely suppressed tumor recurrence and significantly extends tumor-free survival. Inhibition of β-CATENIN-CBP pathways sensitized TICs to IVIG leading to tumor-free survival for three months.

As β-CATENIN + NANOG and YAP1 transactivate the BIRC5 promoter (one of the WNT/β-CATENIN-target genes), we tested whether combinations of these proteins have cooperative transactivation abilities. In p53^−/−^ mouse liver progenitor cell line PIL4, β-CATENIN + NANOG transactivated BIR5 promoter activity (**Supplementary Figure 3**). We next tested whether transducing combinations of these proteins had malignant transformative activities *in vitro*. Co-transduction of β-CATENIN + NANOG resulted in the highest number of colonies, more than triple the amount observed for YAP1 alone (**Figures 5A, B**). This hinted that the knockdown of the major NANOG target gene YAP1 had the most potent effect on HBX and NANOG-mediated anti-tumor effect. BIRC5 was elevated in HCC patients (**Supplementary Figure 4**). BIRC5 is elevated in metastatic patient groups than in the primary HCC site. Elevated BIRC5 patients have lower survival rates than those who have lower BIRC5 expression (**Supplementary Figure 4**). β-CATENIN/YAP1 + NANOG-mediated gene expression have important roles and the results indicated that β-CATENIN and NANOG cooperatively transactivate β-CATENIN–target genes. As such, this group of proteins became of interest for the following intrasplenic cell injection study (**Figure 5C**, Top). Tumor growth with the hepatoblast-transformation in C57Bl/6 mice was studied. These transformed hepatoblasts are sensitive to the growth inhibitory effect of PRC-724 (**Figure 5C**). β-CATENIN and NANOG increased liver:body weight ratio and numbers of tumors in an intrasplenic C57Bl/6 injection model (**Figure 5C**), This anti-tumorigenic effect of *Nanog* and *β-Catenin* silencing on TICs is also observed when TICs are transplanted to the liver via splenic injection in C57BL/6 mice pre-treated with retrosine and post-treated with carbon tetrachloride (**Figure 5C**). Silencing of *Nanog* and *β-Catenin* retards the tumor growth by 50-70% as compared to vehicle-treated group. Silencing of *Nanog* and *β-Catenin* retards the tumor growth by 50-70% as compared to vehicle-treated group. Additionally we used 16 sets of separate Huh7 cell lines transduced with lentivirus vectors expressing different combinations of β-catenin, NANOG, YAP1, HBX, sh-YAP1 or sh-β−CATENIN for intrasplenic injection into C57Bl/6 mice. The levels of *Nanog* and *β-Catenin* were also examined in the tumor tissues collected from the mice treated with the PRC-724 only vs. the reduced tumors in mice with the PRC-724 therapy, demonstrating that the PRC-724 therapy reduced NANOG and β-Catenin/CBP-inducible protein expression in tumors with retarded growth. After 2 months, these animals were scanned for tumor growth using ultrasound and microCT. The HBx + scrambled shRNA (Scr) and HBx + NANOG + Scr groups had the highest tumor burden, while the other groups showed little to no tumor burden (**Figures 5D–F**, **Supplementary Figure 7**, **8**).

### Hepatitis B intravenous immunoglobulin and Hepatitis C intravenous immunoglobulin inhibit proliferation and self-renewal

Based on the previous results, stopping self-renewal of TICs by antagonizing β-CATENIN activity should allow clearance of these weakened TICs due to loss of self-renewal. This could be accomplished with immunoglobulins that capture TICs to clear this cell population in patients or humanized mice harboring human HCCs. TICs may be recognized by intravenous, immunoglobulin obtained from HBV patients since cancer-driver mutation-targeted immune responses are known to be triggered by neoantigens or other TIC-specific cell surface markers. Use of pooled HBV^+^ patient sera (100-200 donors) with or without prior HBIG treatment could be used for this purpose.

**Figure 6:**
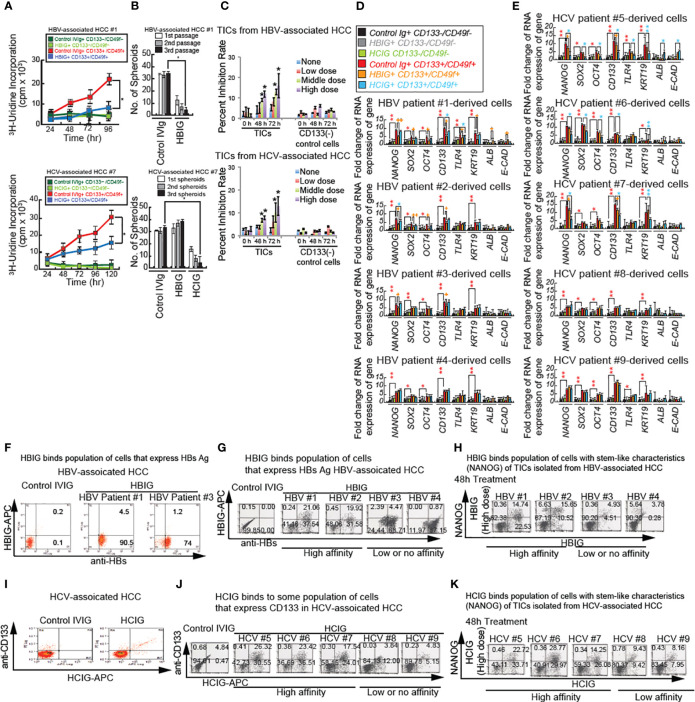
Intravenous immunoglobulin treatment synergistically reduces cancer proliferation and self-renewal ability through inhibition of β-CATENIN. **(A)** Immunoglobulin (isolated from HBV patients: HBIG) treatment reduced stemness and proliferation of TICs *in vitro*. Cultured TICs (CD133^+^/CD49f^+^) and corresponding control cells (CD133^−^/CD49f^−^) from liver tumors 1 and 7 were treated with either IVIG (12.5 mg/mL) or HBIG (5.22 IU/mL) and HCIG (12.5 mg/ml) for different durations and examined for ^3^H-uridine incorporation. **(B)** Spheroid formation in cultured TICs (CD133^+^/CD49f^+^) from liver tumor 1 and 7 were treated with either IVIG (12.5 mg/mL) or HBIG (5.22 IU/mL) for 48 hours. Spheroids on ultra-low adhesion plates underwent serially passages for three times. HBIG treatment significantly inhibited spheroid formation ability. **(C)** MTT assays in TICs treated with HBIG or control IVIG for 48 hours. Freshly isolated and purified TICs, and CD133^-^ control cells were treated with different concentrations of HBIG: low, 0.78 IU/mL (1500 IU in humans); medium, 3.13 IU/mL (6000 IU in humans); high, 5.22 IU/mL (10,000 IU in humans) for 48 or 72 hours. Cell viabilities were determined by MTT assay. (C, bottom) HCIG (Low: 6.25 mg/ml (200 mg/kg in humans), Middle: 9.375 mg/ml (300 mg/kg in humans), High: 12.5 mg/ml (400 mg/kg in humans)). For each case, experiments were carried out in triplicate. Results are expressed as mean ± SD, n = 3, *P < 0.05 vs. respective control. **(D, E)** Cultured control CD133^−^/CD49f^−^ cells and CD133^+^/CD49f^+^ TICs treated with HBIG **(D)**, HCIG **(E)** or control IVIG for 48 hours and examined for stemness, bipotential, and epithelial marker expression. Incubation of control CD133^−^/CD49f^−^ cells with IVIG is shown in black bars and represents baseline levels for the genes tested at 48 hours after treatment. KRT19: CYTOKERATIN19. Stars above the bars indicate statistical significant (*P < 0.05, **P < 0.01). Technical replicates (n = 3) are shown. **(F)** HBIG (Intravenous Hepatitis B Immunoglobulin) binds TICs as analyzed by FACS analyses. TICs were permeabilized and tested for HBs using FACS analysis. Cultured TICs were treated with HBIG or control IVIG for 48 hours. HBIG binding was determined by two-color FACS labeling cells for the immunoglobulin and the proposed target antigen (HBs Ag or E2). **(G)** HBIG (Hepatitis B Immunoglobulin) binds HBs(+) cells as analyzed by FACS analyses. Cultured TICs were treated with HCIG or control IVIG for 48 hours. HBIG binding was determined by two-color FACS labeling cells for the immunoglobulin and the TIC marker CD133. **(H)** HBIG binds NANOG^+^ TICs (stem-like characteristics) by FACS analyses. HBIG can bind to subsets of TICs derived from HCC tumors associated with HBV. Cultured TICs were treated with HBIG or control IVIG for 48 hours and examined for NANOG expression and HBIG binding. For the 0-hour time point, TICs were harvested and incubated with HBIG for 30 minutes and permeabilized and processed for anti-NANOG antibody staining, followed by secondary antibody staining. **(I)** HCIG binds stem-like characteristics (NANOG^+^) TICs assayed by FACS analyses. HCIG can bind to subsets of TICs derived from HCC tumors associated with HCV. **(J)** Cultured TICs were treated with HCIG or control IVIG for 48 hours and examined for CD133 expression and HCIG binding. For the 0-hour time point, TICs were harvested and incubated with HCIG for 30 minutes and permeabilized and processed for anti-CD133 antibody staining, followed by secondary antibody staining. Low affinity: Less than 20% of HBIG or HCIG-bound population. High affinity: More than 20% of IVIG-bound population. **(K)** HCIG binds NANOG^+^ TICs (stem-like characteristics). Cultured TICs were treated with HCIG or control IVIG for 48 hours and examined for NANOG expression and HCIG binding. For 0 h time point, TICs were harvested and incubated with HCIG for 30 min and permeabilized and processed for anti-NANOG antibody staining, followed by secondary antibody staining.

The proliferative activity of TICs was measured by ^3^H-uridine incorporation and spheroid colony formation assays after treatment with HBIG antibodies. Indeed ^3^H-uridine was reduced in CD133+/CD49f+ TICs incubated with HBIG (**Figure 6A**). By contrast, ^3^H-uridine incorporation was unchanged in CD133^-^/CD49f^-^ control cells when incubated with either control IVIG or HBIG (**Figure 6A**).

Incubation of TICs from liver tumor #1 with IVIG did not change spheroid numbers. Whereas significantly fewer spheroids were detected after incubation with HBIG, even with higher passage spheroids (**Figure 6B**). Similarly, incubation of TICs from liver tumor #7 with HBIG led to significantly fewer spheroids (data not shown). In summary, these results showed that HBIG and HCIG treatment reduced proliferation and viability of TICs.

### HBIG or HCIG treatment reduces the viability of TICs

To further evaluate the effect of HBIG on the viability of TICs, TICs were treated with HBIG at different concentrations and were examined by MTT assay. The treatments with HBIG for 48- and 72-hours dose-dependently reduced the viability of TICs (**Figure 6C**, middle dose). Moreover, the viability of TICs was reduced 80%-90% after 72 hours of high concentration HBIG treatment. However, this inhibitory effect of HBIG was not observed for normal peripheral CD133^-^/CD49f^-^ control cells, which were treated with HBIG under the same conditions. These results indicated that the cytotoxic effects of HBIG on TICs were specific. Cytotoxicity assays measured by MTT and colony formation with HBIG or HCIG were further examined against a panel of seven HCC cell lines, mouse TICs, and human TICs isolated from an HBV^+^ patient (see **Supplementary Table 4**, **Figure 1A**, bottom) (28).

### HBIG reduces NANOG expression in patient-derived TICs

To determine if HBIG binds and reduces populations/oncogenesis of TICs isolated from human patients, the antibody-binding activity was examined. For TICs isolated from six HBV patients, NANOG expression was detected in > 20% of cells. The isolated TICs showed an undifferentiated morphology and expressed stemness markers (CD133 and NANOG) as determined by qRT-PCR (**Figure 6D**). After 2 days of HBIG treatment, TICs lost stem-like characteristics (CD133, SOX2, OCT4, and NANOG), liver progenitor cell markers (CYTOKERATIN: KRT19), and epithelial cell marker (E-CAD) or were killed off. As shown in **Figure 6D**, CD133^+^/CD49f^+^ TICs expressed the bipotential marker CK19 as well as the stemness markers CD133, NANOG, SOX2, OCT4, and TLR4 at levels 2-6-fold higher than control CD133^-^/CD49f^+^ cells. These cells were affected by coincubation with HBIG. By contrast, control CD133^-^/CD49f^-^ cells expression levels for KRT19, CD133, NANOG, SOX2, OCT4, and TLR4 were independent of IVIG (immune serum from people with HBV^+^ or HCV^+^ liver disease) or HBIG incubation. A significant decline of expression levels for NANOG, SOX2, OCT4, and CD133 was detected in HBIG with high affinity for CD133^high^ cells from HBV associated tumors (patients 1 and 2) after HBIG incubation. Whereas TLR4 and KRT19 underwent significant reduction in TICs from patient #1. No significant differences for SOX2, OCT 4, TLR4, and KRT19 were detected in the low-affinity TICs (patients #3 and #4). NANOG and CD133 expression was, however, significantly reduced in the low affinity TICs from patient 3 but not in the TICs from patient 4. Expression of ALB and E-CAD were generally not remarkably different between CD133^+^/CD49f^+^ TICs and control CD133^-^/CD49f^+^ cells and did not change when incubated with either IVIG or HBIG. In high-affinity TICs derived from patients 1 and 2, negligible amounts of cells expressed stemness marker NANOG after HBIG treatment (**Figure 6D**). These results indicated that HBIG reverted cells to the non-TIC phenotype or simply killed off this population. Similarly, NANOG, SOX2, OCT4, CD133, and KRT19 expression were significantly reduced after HCIG incubation (isolated from serums of 100-200 HCV patients) in high affinity TICs from HCV associated tumors (patient 5, 6, and 7), whereas expression was not altered significantly in low affinity TICs (**Figure 6E**).

### HCC TIC binds patient-derived intravenous immunoglobulin

HBIG binds hepatitis B antigens (such as HBs or HBc)-expressing hepatocytes, including transformed hepatocytes (39) and (**Figure 6F**) possibly HBV-associated HCC cells. To determine if isolated cell populations express HBV viral antigens (HBs), TICs were permeabilized and tested for HBs using FACS analysis. Cultured TICs were treated with HBIG (Nabi-HB) or control IVIG (Bivigam) for 48 hours (**Supplementary Table 4**). HBIG binding was determined by two-color FACS labeling of cells for the Hepatitis B and Hepatitis C immunoglobulin (HBIG and HCIG, respectively) and the proposed target antigen (Hbs Ag or E2). In TICs from HBV-associated HCC samples, >70% of TICs were positive for HBs antigen (**Figure 6G**). HBIG also bound to subsets of TICs derived from HCC tumors associated with HBV infection (**Figure 6G**). The HBIG binding activity (antigen density) was higher with 16.82% and 16.95% of positively staining cells for TIC #1and #2 (high-antigen TICs) as compared to TIC#3 and #4 (low antigen TICs), which exhibited low or no double positive cells (**Supplementary Table 5**). By comparison only 8-15% of cells derived from patients #7, #8 and #9 were Nanog positive. CD133^+^ population that were isolated from HCV-associated HCCs bound HCIG (**Figures 6I, J**). Similarly, in TICs derived from patient #5 and #6 but not #7 negligible amounts of cells expressing NANOG were detected after Hepatitis C intravenous immunoglobulin (HCIG) treatment compared to the control time point. No difference or a slight increase of cells expressing NANOG was found for the low antigen TICs from #8 and #9 (**Figures 6H, K**). These results indicated that HBIG and HCIG bound TICs isolated from HBV- or HCV-associated HCCs, respectively.

### HBIG treatment reduces TICs in xenograft models

To validate the antitumorigenic ability of HBIG or HCIG *in vivo* in NOG mice, we transplanted the NANOG^+^/CD133^+^/CD49f^+^cells with or without HBIG and determined the effect of PRI-724 on tumor growth. To determine if HBIG or Biotest-HCIG inhibits tumorigenic potential and growth, human TICs were subcutaneously xenografted into immunocompromised NOG mice in the presence or absence of HBIG or Biotest-HCIG treatment (HBIG doses are shown in **Supplementary Tables 7**, **8**).

**Figure 7:**
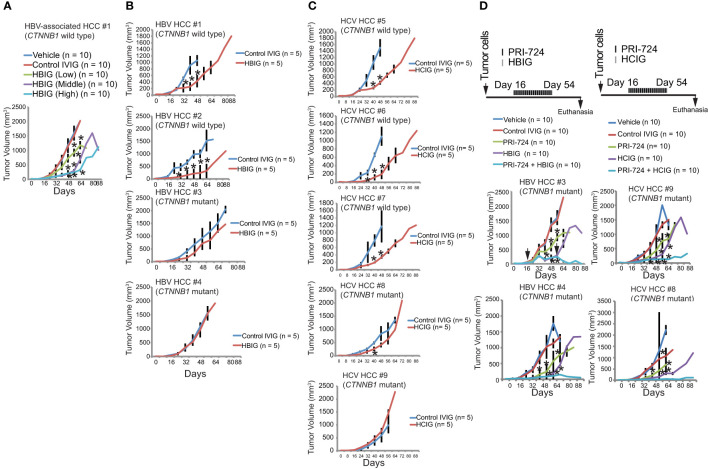
Intravenous immunoglobulin (isolated from HBV or HCV patients) binds HBs Ag (+) cells and NANOG^+^ TICs. **(A)** Tumor growth in freshly isolated and purified TICs, and CD133(−) control cells treated with different concentrations of HBIG: low, 1.56 IU/mouse; 5 μL (1500 IU in humans); medium, 6.25 IU/mouse: 20 μL (6000 IU in humans); high, 10.4 IU/mouse: 33.3 μL (10,000 IU in humans) or 25 mg/mouse (equivalent to 400 mg/kg in humans). Results are expressed as mean ± SD, n = 10, *P < 0.05 vs. respective control. **(B)** Freshly isolated and purified TICs, and CD133^−;^ control cells treated with different concentration of HBIG High-dose: 10.4 IU/mouse: 33.3 μL (10,000 IU in humans). Results are expressed as mean ± SD, n = 10, *P < 0.05 vs. respective control. *CTNNB1*: β-CATENIN-coding gene. Mutations were detected in exon 3 of CTNNB1 in serin and/or threonine residues that stabilizes β-CATENIN protein. **(C)** HCIG treatment reduces tumor growth. Freshly isolated and purified TICs, and CD133^-^ control cells were treated with different concentration of HCIG (12.5 mg/mouse (equivalent to 200 mg/kg in humans), 18.75 mg/mouse (equivalent to 300 mg/kg in humans), 25 mg/mouse (equivalent to 400 mg/kg in humans). Results are expressed as mean ± SD, n=10, *P<0.05 vs. respective control. **(D)** Tumor growth in tumor-bearing mice administered with PRI-724 and HBIG (Left) or HCIG (Rightm) for HBV-associated tumors and HCV-associated HCCs. HBIG and PRI-724 treaments were combined and tumor growth was monitered.

We observed that HBIG or HCIG pretreatment significantly decreased the tumor-seeding ability of TICs relative to drug pretreatment for both human TICs. These findings indicated that TICs within HCC populations are resistant to conventional chemotherapy but sensitive to treatment with HBIG or Biotest-HCIG. While palpable tumors developed in vehicle-treated mice within 1.5 weeks, drug treatment delayed palpable tumor formation by five weeks. Subsequent tumor sizes in HBIG-treated animals were reduced relative to tumors in vehicle-treated animals (**Figures 7A, B**). Tumor size reduction relative to vehicle-treated controls were comparable for drug-treated mice, the latter cohort exhibited a reduced tumor size at later time points.

### Inhibition of β-CATENIN and HBIG treatment synergistically eradicates xenograft tumors

Previous studies have shown that an intrinsic β-CATENIN signaling prevents antitumor immunity (40, 41). To examine the status of *CTNNB1* (β-CATENIN coding gene), we sequenced the HBIG-resistant TICs. Although HBIG treatment did not completely reduce the derived TICs, the major HCC driver genes were nonetheless sequenced. The drug insensitive population may be due to the fact that HBsAg was not expressed on those cells. The HBIG-resistant tumors harbored a *CTNNB1* mutation in exon 3 (mutation hotspot region) that stabilized β-CATENIN protein (**Figure 7B**). *CTNNB1* mutations were consistently detected in HBIG-resistant TICs. *CTNNB1* mutations were consistently detected in HCIG-resistant TICs isolated from HCV-associated HCC patients (**Figure 7C**). To overcome constitutively-active β-CATENIN mutation, PRI-724 treatment was combined with HBIG or HCIG for xenograft mouse tumor preclinical trials. Therefore, we tested whether conjunctive treatment with HBIG and the WNT/CBP-pathway inhibitor had synergistic tumor killing effects. Treatment with WNT/CBP inhibitor (PRI-724) and HBIG significantly reduced TIC-derived tumor growth (**Figure 7D**, left). Conjunctive treatment with WNT/CBP inhibitor (PRI-724) and HCIG significantly reduced TIC-derived tumor growth derived from HCV-associated HCC patients (**Figure 7D**, right). These results indicated that the *CTNNB1* mutation status could serve as a biomarker for efficacy of this conjunctive treatment. Tumor growth was inhibited and tumor size remained stable, indicating that β-CATENIN targeting synergized tumor killing effects and improved the survival of tumor bearing mice.

## Discussion

We demonstrated that HBIG binds TIC populations that express HBsAg or E2 Ag and reduces stem-like characteristics (CD133), proliferation and viability of TICs. HBIG causes significant but partial regression of liver tumors in tumor-bearing mice. Clinical evidence revealed development of resistance (3 of 51; 5.8%) to these HBIG treatments in HCC patients who underwent liver transplantation in a clinical trial (42). The proposed mechanisms by which HBIG reduces stemness marker expression in qRT-PCR experiments are not yet understood.

How does HBIG bind to chronically HBV infected patient samples? HBIG or HCIG may bind circulating tumor cells (CTC) more efficiently rather than tumor mass to capture and eliminate the possible metastatic tumor spread or recurrence to inactivate before CTCs extravasate and colonize in distal organs or intrahepatic metastasis. Efficient binding of HBIG or HCIG to disseminated cells from primary HCCs rather than the tumor mass itself would be an ideal characteristic to capture all CTCs before they reach distal organ or intrahepatic HCC spread.

The mechanism by which Ig binding reduces expression of NANOG is unknown. The first possible explanation is that antibody binding to the cell surface inhibits cell aggregation or cell-cell interaction to block self-renewal ability, since growing TICs have an aggregated phenotype as opposed to quiescent TICs (**Figure 5G**). The other possible explanation is that binding to the cell surface may stimulate an unspecified differentiation signaling pathway. Future investigation is warranted to resolve this.

The doses of HBIG used in this study are well tolerated as shown in chimpanzee and human trials (43), and reflect the levels that are clinically administered to human HCC patients (44). The mechanism by which HBIG prevents metastatic spread may also be multifactorial (**Supplementary Table 9**) (45–47). In theory, HBIG can affect each step in the process of metastatic spread, from angiogenesis to direct killing (lysis) of the malignant cells (45).

Here we have shown that HBx is crucial for regulating the β-CATENIN and NANOG levels in HBV transgenic mice. HBx-negative mice showed little β-CATENIN and NANOG expression, but conversely those mice that had a full-length HBV genome with HBx had much higher expression levels. Mice also showed significantly increased levels of nuclear localization of β-CATENIN and NANOG in the presence of HBx. Despite HBx’s ability to regulate both β-CATENIN and NANOG to transactivate EZH2, it seems that an initiation event (DEN treatment) is required before both components move into the nucleus and regulate their downstream targets (**Supplementary Table 3**).

The roles of several markers tested in the study will serve as companion diagnostic tools to stratify responders and non-responder HCC patient groups and better treat HCC patients by HBIG or HCIG administration.

To summarize, HBx can regulate β-CATENIN and NANOG, which work together with YAP1, leading to the development of HCC potentially through the activation of BIRC5. HBx itself can induce EZH2 expression through the NANOG and STAT3 elements in the EZH2 promoter region. Based on preliminary data, YAP1 interaction with β-CATENIN and NANOG seems to play an important role, as removing β-CATENIN from the HBx and HBx + NANOG models decreased colony formation and tumor incidence. Further studies will be required to correctly pinpoint the connections between these pathways, as well as using dual-lineage tracing to determine exactly from what cells HCC is derived. Elucidating the major pathways required for HCC development can allow development of drugs to target those pathways to provide better treatment and prognosis for late-stage HCC patients.

## Materials and methods

Detailed experimental procedures and reagents were summarized in **Supplementary Tables 1**, **2**.

### Transgenic mice

Transgenic mouse liver samples from DEN- Tg05, DEN^+^ Tg05, Tg31, Tg08, and Tg04 were kindly provided by Dr. James Ou’s laboratory at the University of Southern California (**Supplementary Table 3**). Mice were anesthetized by isoflurane (1-4%) [inhaled]. For end-point studies, mice were anesthetized with Ketamine (80-100 mg/kg) and Xylazine (5-10 mg/kg), followed by cardiopuncture-based euthanasia process.

### Isolation of human bipotent TICs

Differentiated liver tumors of patients who had HCC with chronic HBV or HCV infection were processed to make primary cultures and to isolate TICs that were rigorously validated in previous studies in our lab (17, 42, 43). A total of 28 TIC isolates were obtained over two years. Four TIC isolates out of nine primary HCCs were usable for the associated with HBV or HCV studies (**Supplementary Table 4**).

Accession number: ENCSR000ARI

Assay: ChIP-seq (TF ChIP-seq); Target: EZH2; Biosample summary. Homo sapiens HepG2; Biosample Type: cell line.

Experiment summary for ENCSR000ARI

encodeproject.org

https://www.encodeproject.org, experiments

Accession number: Sample GSM4133289

H3K27Ac_rep1_HepG2_ChIPsep

https://www.ncbi.nlm.nih.gov/geo/query/acc.cgi?acc=GSM4133289

RELACS HepG2 H3K27ac

GSM3019929

RELACS HepG2 H3K4me3

GSM3019930

Nov 16, 2010 — Homo sapiens HepG2. Biosample Type: cell line. Replication type: isogenic. Description: ChIP-seq on human HepG2 EZH2. Nucleic acid type: DNA.

### Immunoglobulin preparations

Hepatitis B immunoglobulin (HBIG; HBIG™) and control 10% intravenous immunoglobulin (IVIG; BIVIGAM™) antibody preparations were obtained from Biotest Pharmaceuticals Corporation.

### Antibody staining and FACS analysis

Cultured TICs were treated with HBIG, HCIG or control IVIG for 48 hours at different concentrations and examined for HBIG or HCIG binding as well as NANOG expression.

For the 0 h time point, TICs were harvested and incubated with HBIG or HCIG for 30 min. For HBIG and HCIG binding, we pre-labeled HCIG/HBIG and HBs Ag/E2 with APC and PE before FACS. Cells were permeabilized and processed for anti-NANOG antibody staining, followed by secondary antibody staining.

### Quantification of immunohistochemistry staining

To quantify the levels of β-catenin and NANOG staining in the mouse tissue samples, an immunoreactivity score was calculated, as per Koomagi et al. (22). The first of two variables calculated was the intensity of staining, scored on a scale from 0 to 3 (0 = negative staining; 1 = weakly positive; 2 = moderately positive; and 3 = strongly positive). The second variable determined was the extent of distribution of nuclear positive cells, scored on a scale from 0 to 4 (0 = negative; 1 = positive staining in 0.01–1.0% of the cells; 2 = 1.01–2.0%; 3 = 2.01–3.0%; and 4 = > 3.0% positivity). To calculate the immunoreactivity score, the intensity of staining score was multiplied by the extent of distribution score. Immunoreactive scores ≥ 2 were considered positive staining. Images were evaluated by two independent investigators, and their two scores were averaged.

### Antibody staining and FACS analysis

To determine if HBIG antibodies bind to propagated tumor stem cells, FACS analysis was used. Cultured TICs were treated with HBIG or control IVIG for 48 hours at different concentrations and examined for HBIG binding as well as NANOG expression. For the 0-hour time point, TICs were harvested and incubated with HBIG for 30 minutes. For HBIG binding, HBIG and HBsAg were pre-labeled with APC and PE for 30 minutes before FACS. Cells were permeabilized and processed for anti-NANOG antibody staining, followed by secondary antibody staining.

### *In vivo* xenograft assay in NOG mice

TICs (50,000 cells) were injected at day 0. HBIG was injected intraperitoneally. Control mice were injected with either PBS or HBIG at indicated doses, once a week before TIC injection and from day +7 to day +88 as previously described (44). Tumor volume was determined from green fluorescent protein (GFP)−positive TICs. This was performed by functional bioluminescent imaging every eight days until day 88. As the treatment was initiated at one week before tumor injection, all treatments ended at day 88; all surviving mice were euthanized at that time. After euthanasia, we determined the tumor volumes and dimensions.

### Western blot and immunohistochemistry

Transgenic mouse tissue samples were prepared for protein isolation in RIPA buffer as previously described (17). Protein samples (30 µg each) were resolved by SDS polyacrylamide electrophoresis and transferred via semi-dry transfer to nitrocellulose (GE Water and Process Technologies). For immunoblotting, the primary antibodies used were mouse anti-active-β-catenin (1:5000, Santa Cruz Biotech), rabbit anti-NANOG (1:1000, Abcam), and mouse anti-β-actin (1:1000). The secondary antibodies used were goat anti-mouse horseradish peroxidase conjugated secondary antibody (HRP; 1:10000, Santa Cruz Biotech) and goat anti-rabbit HRP (1:10000, Santa Cruz Biotech). The blots were exposed to X-ray film and then processed in an X-ray film developer (Konica Minolta SRX-101A) and quantified using ImageJ v.3.91 (http://rsb.info.nih.gov/ij). For immunohistochemistry, the primary antibodies used were mouse anti-β-catenin (1:250, Santa Cruz Biotech) and rabbit anti-NANOG (1:250, ab80892, abcam). Secondary antibodies were conjugated to HRP and developed using 3,3’-diaminobenzidine with hematoxylin (Invitrogen) or goat anti-mouse fluorescein isothiocyanate (FITC; 1:250, Jackson ImmunoResearch Lab) and donkey anti-rabbit R-Phycoerythrin (1:250, Jackson ImmunoResearch Lab), depending on the application.

### Plasmids

Plasmids used were pBABE-YAP1 (#15682, Addgene), pMXs-NANOG (#13354, Addgene), and pBABE- β-catenin S33Y HA (courtesy of Dr. Been Ze’ev, the Weizmann Institute, Rehovot, Israel). pCMV-HBx HA (courtesy of Dr. Jing-Hsiung Ou, University of Southern California, Department of Molecular Microbiology and Immunology) was also used.

### Statistical considerations

All experiments, excluding the Western blot analysis, were carried out in triplicate. The presented data were the averages of three readings ± the standard deviation. Statistical significance was determined from a *P*-value < 0.05 and was carried out using a two-tailed unpaired Student *t*-test.

## Data availability statement

The original contributions presented in the study are publicly available. This data can be found here: Experiment summary for ENCSR000ARI encodeproject.org https://www.encodeproject.org ›experiments Accession Number: Sample GSM4133289 H3K27Ac_rep1_HepG2_ChIPsep https://www.ncbi.nlm.nih.gov/geo/query/acc.cgi?acc=GSM4133289 RELACS HepG2 H3K27ac GSM3019929 RELACS HepG2 H3K4me3 GSM3019930.

## Ethics statement

Ethical approval was not required for the studies on humans in accordance with the local legislation and institutional requirements because only commercially available established cell lines were used. The animal study was approved by institutional animal care and use committee (IACUC). The study was conducted in accordance with the local legislation and institutional requirements.

## Author contributions

CN, KM, and CC conceived of the study. MN, CN, KM, DK, RM obtained the data. PF, and LM performed the immunostaining data analysis, provided drugs. LM, PF, JO, LS provided HCC tissues. J-HO provided HBV transgenic mouse tissues. KM, MN, CN, SMT, and CC conducted the data analysis and drafted the report. All authors contributed to the article and approved the submitted version.
