# Supplementary material for: β-CATENIN stabilizes HIF2 through lncRNA and inhibits intravenous immunoglobulin immunotherapy
Source: Front Immunol. 2023 Sep 8;14:1204907. doi: 10.3389/fimmu.2023.1204907 (PMC10516572; doi:10.3389/fimmu.2023.1204907)
Supplement: Supplementary file 1 [file DataSheet_1.docx]

**SUPPLEMENTAL INFORMATION:**

**This supplement contains:**

**Supplemental Figures**

- **Suppl. Fig. 1. H & E staining images of liver sections from control DEN- Non-Tg and DEN- Tg05 tissue samples.**
- **Suppl. Fig. 2. Elevated levels of β-catenin and NANOG co-localize in the nucleus in transgenic mouse liver tissue.**
- Suppl. Fig. 3. BIRC5-Luciferase assay carried out in mouse liver progenitor cells, PIL-4.
- Suppl. Fig. 4. Data from *in silico* analysis using The Oncomine^TM^ platform.
- **Suppl. Fig. 5. Expression of pSMAD2/3 proteins in liver diseases associated with HBV.**
- **Suppl. Fig. 6. Expression of IMP3 (IGF2BP3) proteins in liver diseases associated with HBV.**
- **Suppl. Fig. 7. Expression of TLR4 proteins in liver diseases associated with HBV.**
- **Suppl. Fig. 8. Expression of NANOG proteins in liver diseases associated with HBV.**
- **Suppl. Fig. 9 LncRNA *β-CatM* positively regulates EZH2-bound target genes.**
- **Suppl. Fig. 10. HBx in the presence or absence of NANOG expression produced highest tumor burden, while the other groups showed little to no tumor burden in intrasplenic injection into C57Bl/6 mice.**

**Supplemental Table**

- **Supplemental Table 1. KEY RESOURCES TABLE (including Immune Globulin Preparations)**
- **Supplemental Table 2. Primers.**
- **Supplemental Table 3. In vivo oncogenic analysis of HBV Tg mouse model**
- **Supplemental Table 4. TICs isolated from HBV-associated human HCC specimens.**
- Supplemental Table 5. Summary of experiments for FACS studies of bindings between TICs and HBIG for the MTT or spheroid assays.
- **Supplemental Table 6. Cytotoxicity of PRI-724 in a panel of seven HCC cell lines and two TICs.**
- **Supplemental Table 7. Breakdown of mice for therapy**
- **Supplemental Table 8. Summary of experiments for in vivo efficacy studies of HBIG in TICs engrafted in immunocompromised mice (10 mice per dose level)**
- **Supplemental Table 9. Proposed mechanisms for anti-tumorigenic effects of IVIG**

**Supporting Materials and Methods**

**Supporting References**

**Supplemental Information**

**
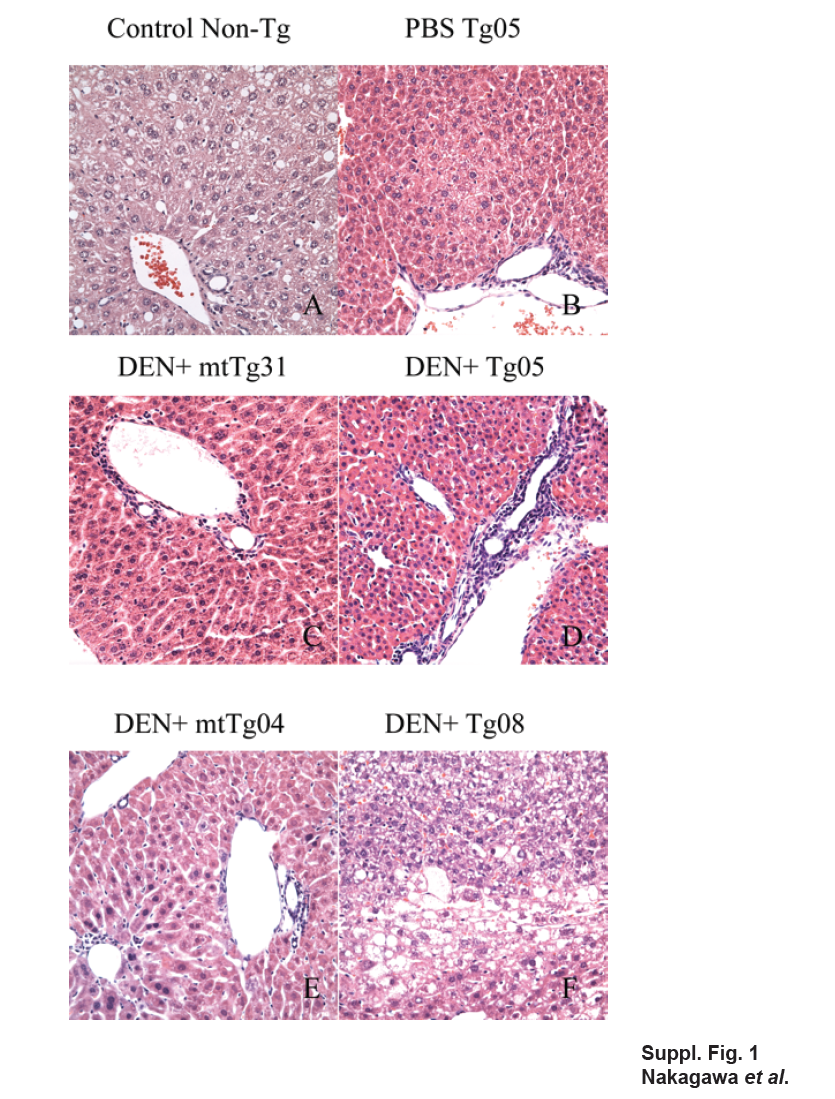
**

**Suppl. Fig. 1. H & E staining images of liver sections from control DEN- Non-Tg and DEN- Tg05 tissue samples.**

H&E staining shows morphological changes in tissue in the presence or absence of HBx and DEN. (A) Control diet, non-Tg sample has normal morphology. (B) wt-HBV genome without DEN injection shows normal phenotype. (C) HBx(-) HBV genome shows normal phenotype. (D) wt-HBV with DEN injection shows expansion of cells near the portal triad. (E) HBx(-) sample shows normal phenotype. (F) wt-HBV with DEN shows tumor mass as well as fatty liver change and some slight dysplasia.

**
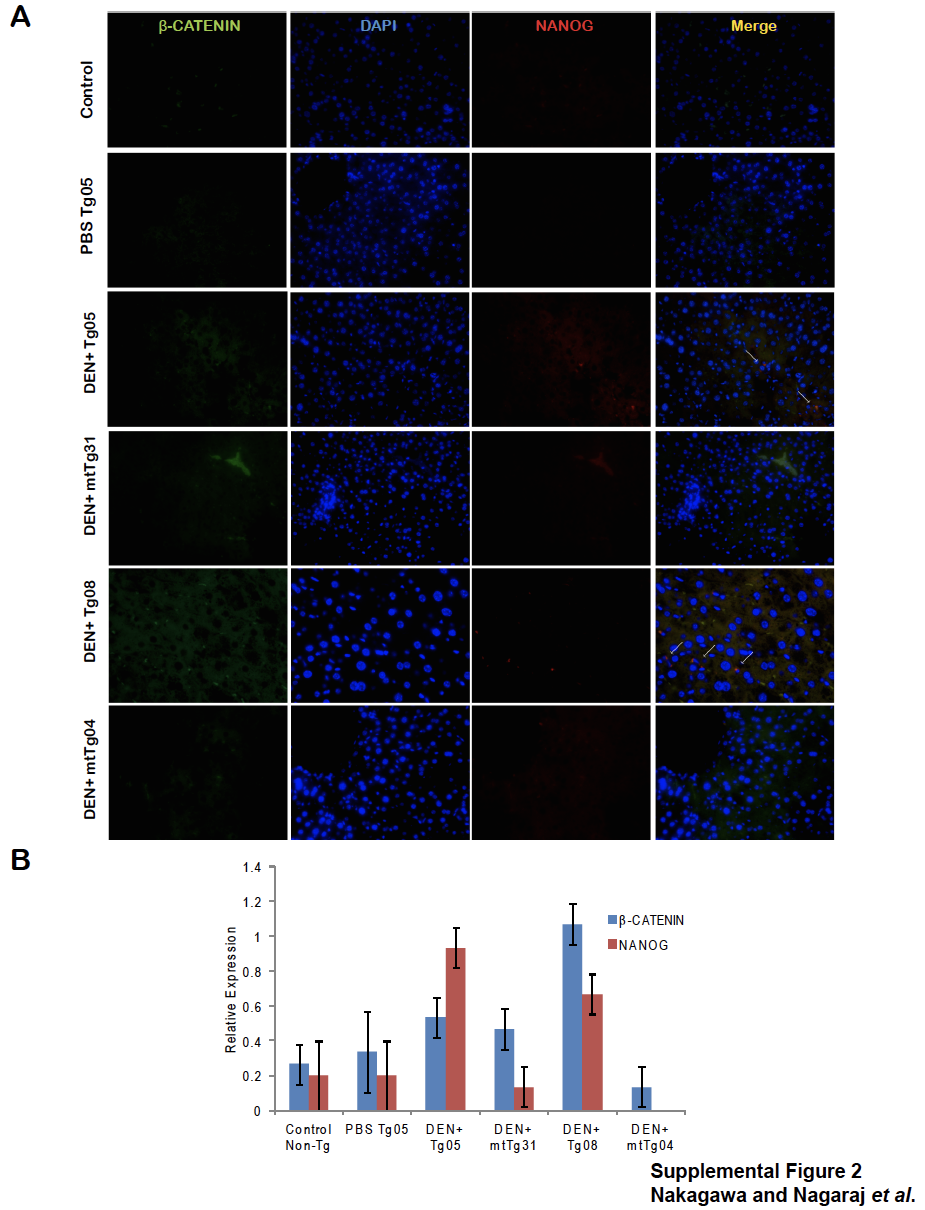
Suppl. Fig. 2. Elevated levels of β-CATENIN and NANOG co-localize in the nucleus in transgenic mouse liver tissue.** Immunofluorescent staining of β-CATENIN [^2^](#_ENREF_2) and NANOG (Rho) using 400x magnification. Nuclear fluorescence was counted in 10 high-power fields and the average was taken (bottom). Each of the samples was double-stained with β-catenin and NANOG antibodies, followed by secondary antibodies with FITC, Rho, or DAPI. Images were visualized through a 40× objective and a 10× eyepiece, resulting in 400× magnification. Removal of HBx significantly reduced NANOG expression levels in both mtTg31 and mtTg04. Only the set Tg08/mtTg04 showed significantly decreased β-catenin expression. * *P* value < 0.05 by two-tailed, unpaired, Student *t*-test.

**
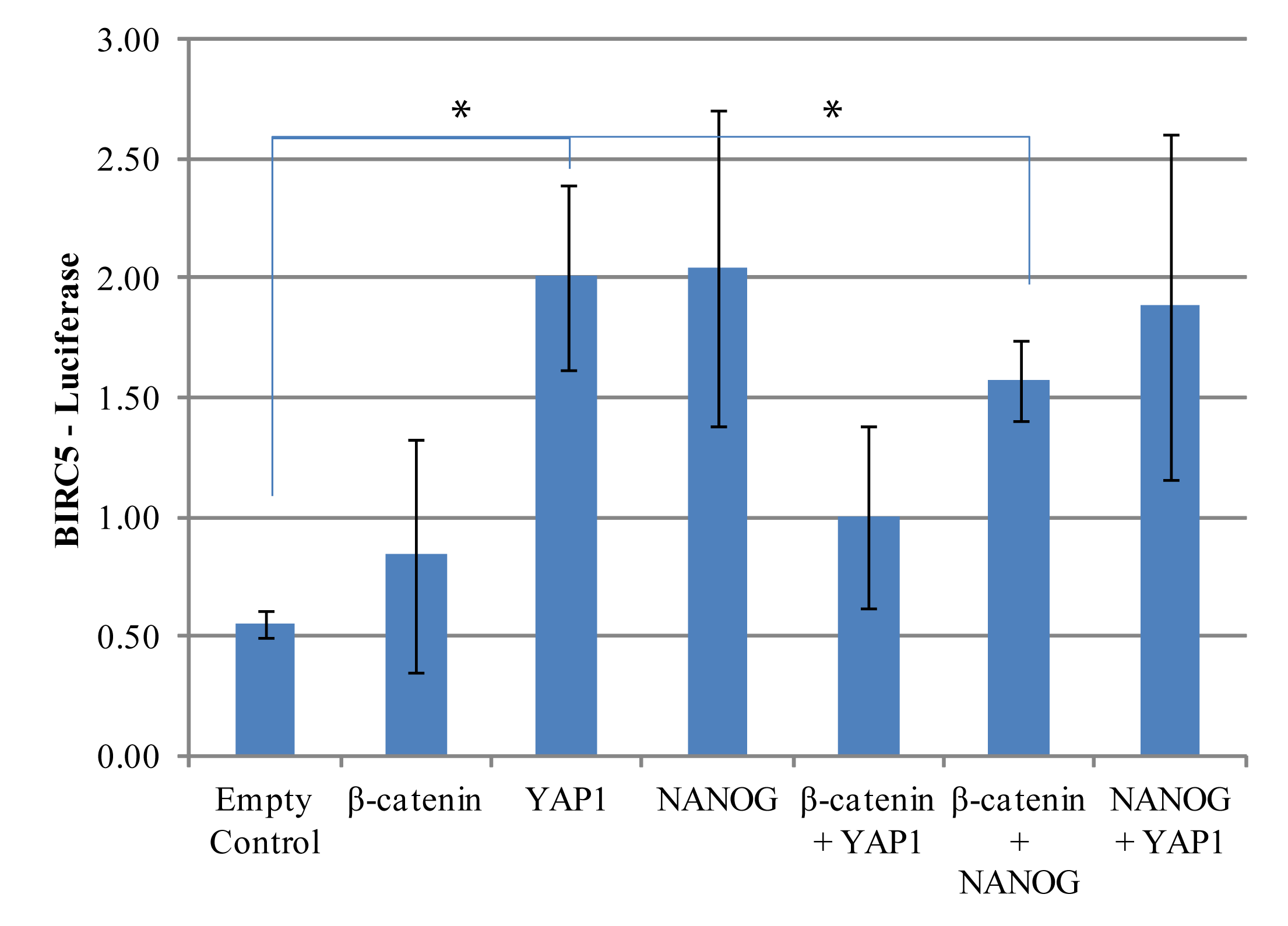
**

Suppl. Fig. 3. BIRC5-Luciferase assay carried out in mouse liver progenitor cells, PIL-4.

Expression vectors were transiently transfected into PIL-4 cells and the luciferase measurements were taken 48 hours after initial transfection. The BIRC5-luciferase readings were standardized to the SV40-Renilla Luciferase. YAP1 and β-catenin + NANOG were the only groups that showed a significant increase in BIRC5 promoter activity when compared to the empty control vector. The * is p-value < 0.05, a two-tailed, unpaired, Student’s t-test.

**
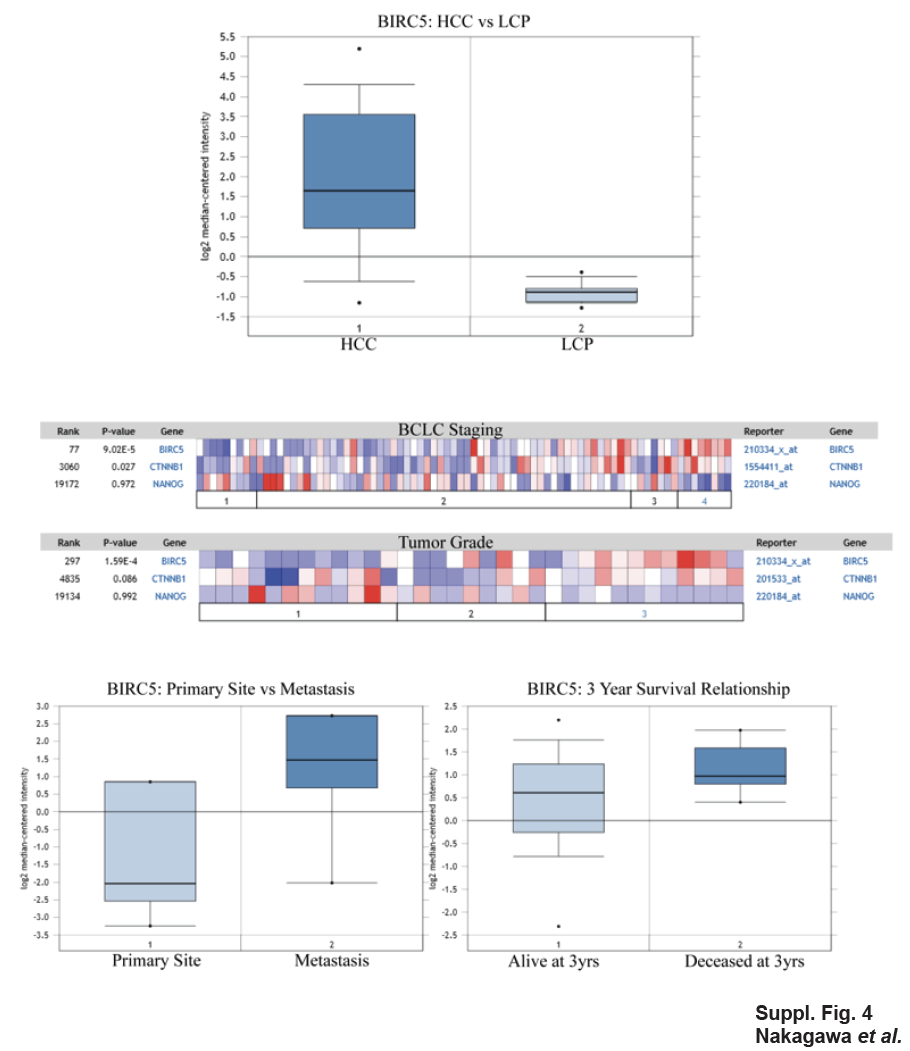
**

Suppl. Fig. 4. Data from *in silico* analysis using The Oncomine^TM^ Platform (Life Technologies, Ann Arbor, MI).

BCLC staging is (1) Stage 0, (2)[^1^](#_ENREF_1) Stage A, (3) Stage B, (4) Stage C. Tumor grade is scored as (1) well differentiated, (2) intermediately differentiated, (3) poorly differentiated. HCC vs LCP (hepatocellular carcinoma vs. liver cancer precursors) (cirrhosis and fibrosis) BIRC5 levels were seen to be more highly expressed in HCC and not in cell precursors to cancer (Data from Wurmbach et al., 2007). Similarly, we see that BIRC5 expression is more prominent at later stages of cancer shown by BCLC (Data from Chiang et al., 2008) and tumor grade (Data from Wurmbach et al., 2007). BIRC5 expression levels were higher in metastasis sites when compared to primary sites (Data from Liao et al., 2008). This has implication for its impact on the establishment of a new tumor location. Also patients with elevated BIRC5 levels had a lower chance of living after 3 years from diagnosis (Data from Hoshida et al., 2009). All data presented on BIRC5 has a p-value < 0.05.


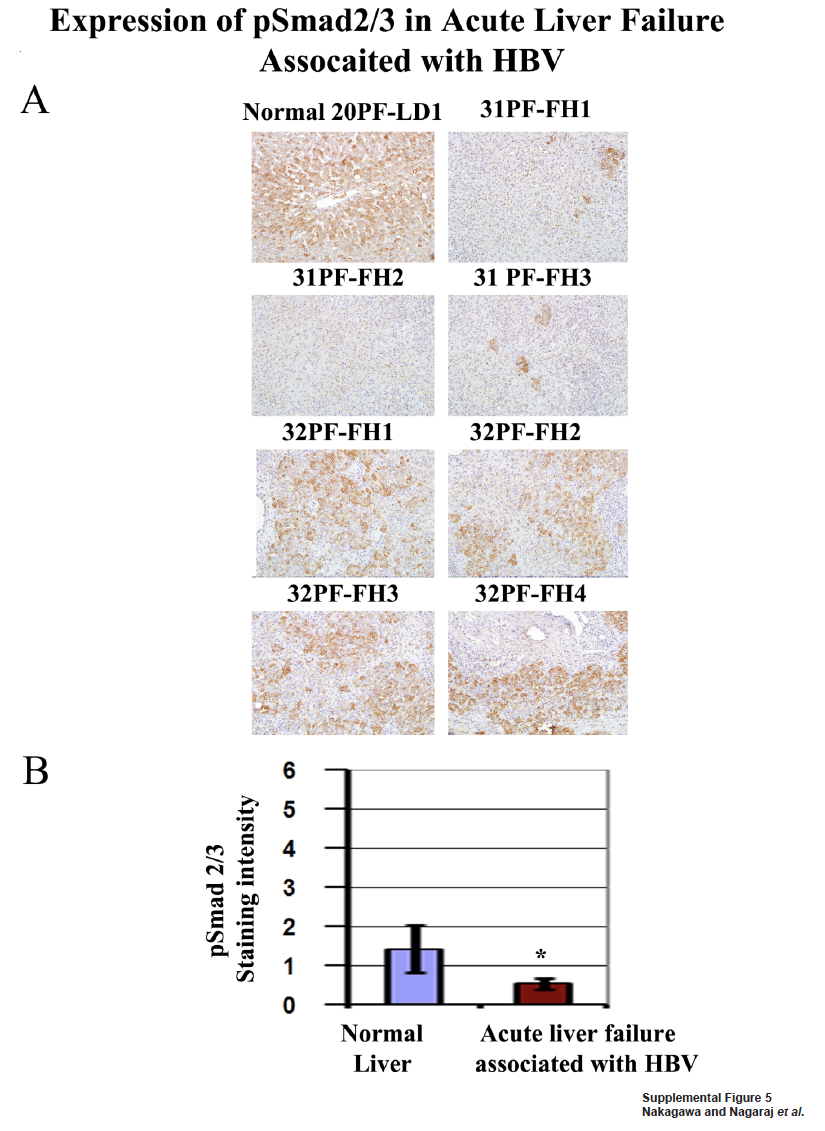


**Suppl. Figs. 5. Expression of pSMAD2/3 proteins in liver diseases associated with HBV.**

pSmad2/3 was down regulated in liver tumors in comparison to normal liver.

**
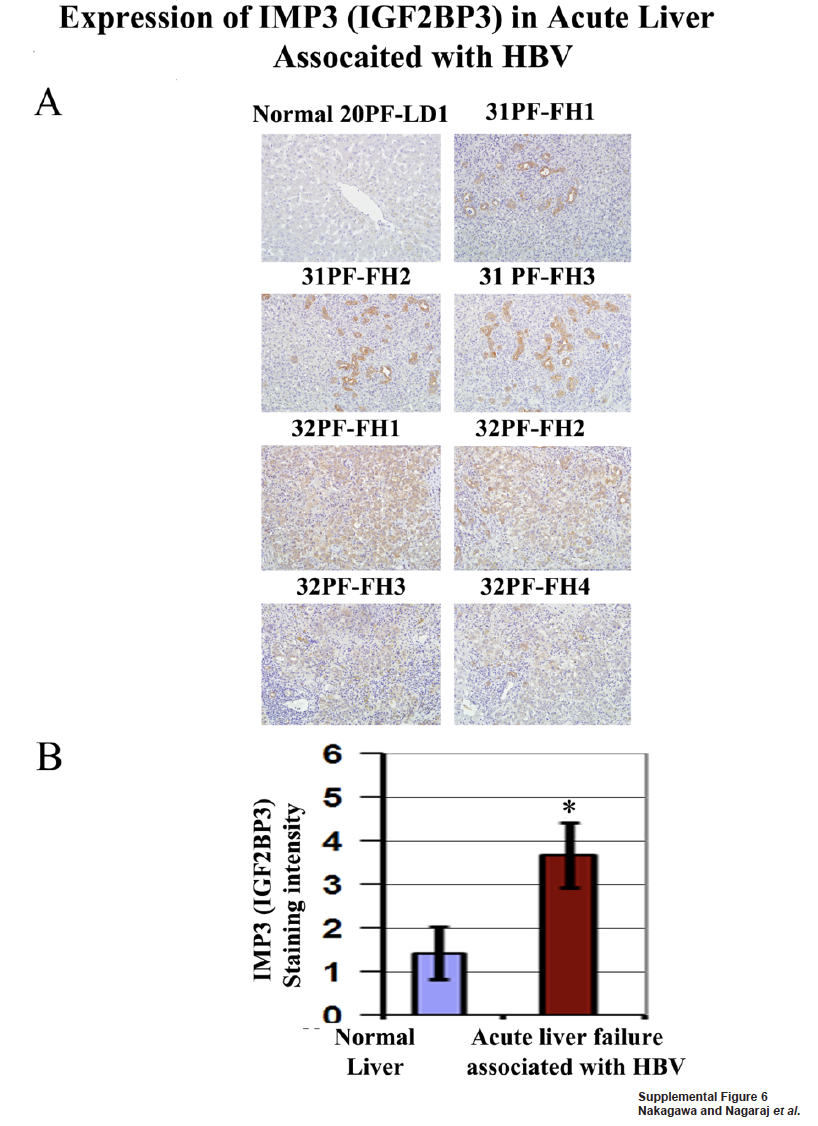
Suppl. Fig. 6. Expression of IMP3 (IGF2BP3) proteins in liver diseases associated with HBV.**

Significant increase in IMP3 (IGF2BP3) cytosolic expression was observed (P<0.05) in liver tumors in comparison to normal liver.

**
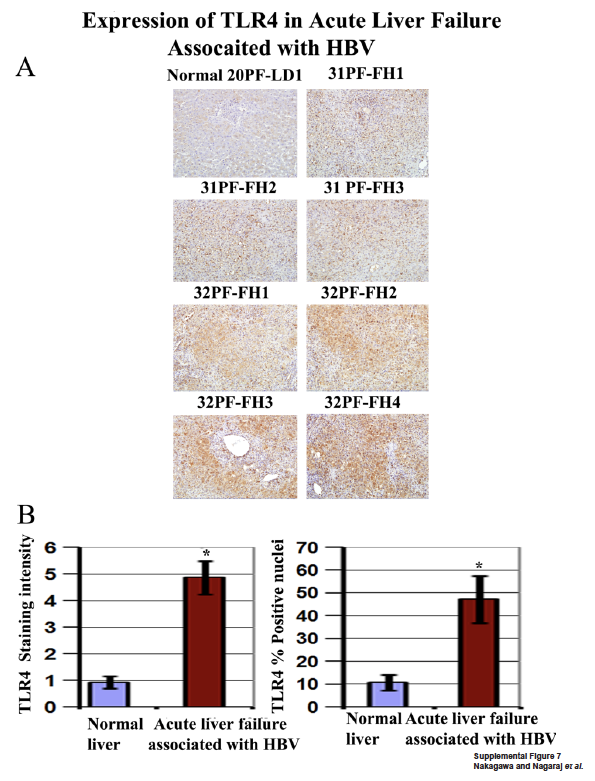
**

**Suppl. Fig. 7. Expression of TLR4 proteins in liver diseases associated with HBV.**

Significant increase in cytosolic TLR4 expression was observed (P<0.05) in liver tumors in comparison to normal liver. Our data further showed a significant increase in nuclear expression of TLR4 in liver tumor samples in comparison to normal liver (P<0.05).

**
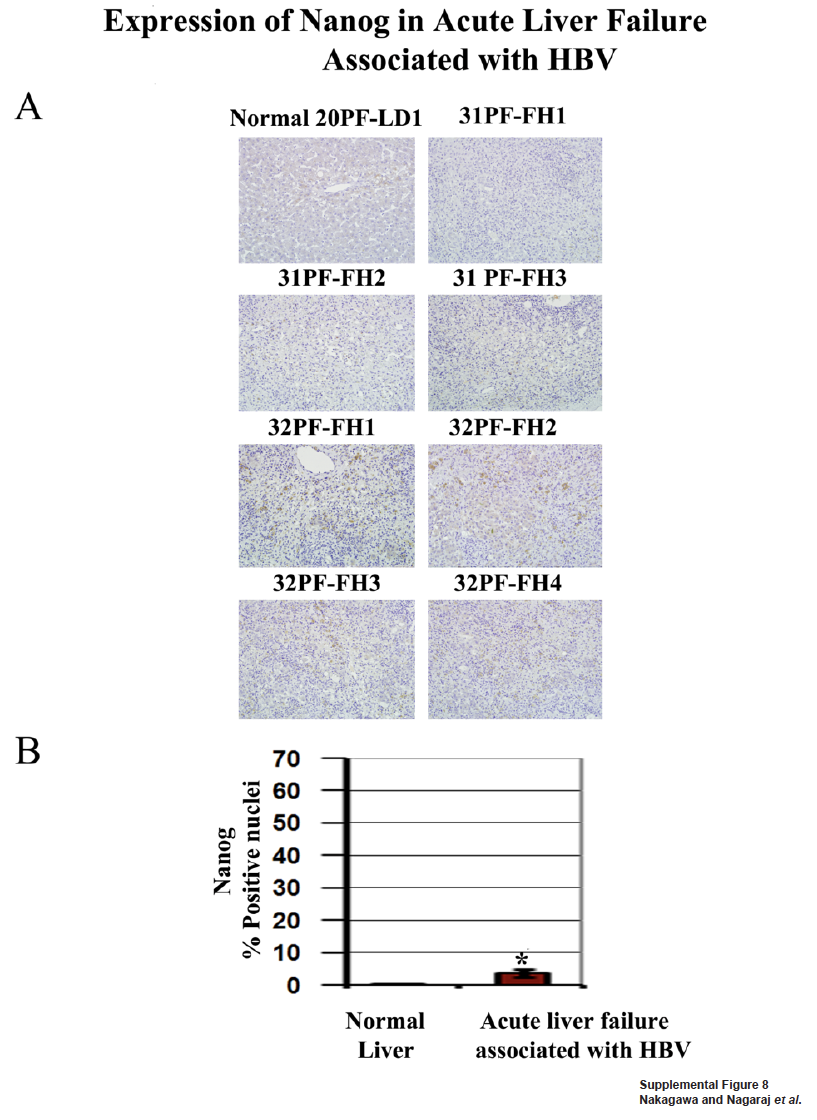
Suppl. Fig. 8. Expression of NANOG proteins in liver diseases associated with HBV.**

Significant increase in NANOG expressions was observed (P<0.05) in liver tumors in comparison to normal liver. Our data further showed significant increase in nuclear expression of NANOG in liver tumor samples in comparison to normal liver (P<0.05).

**
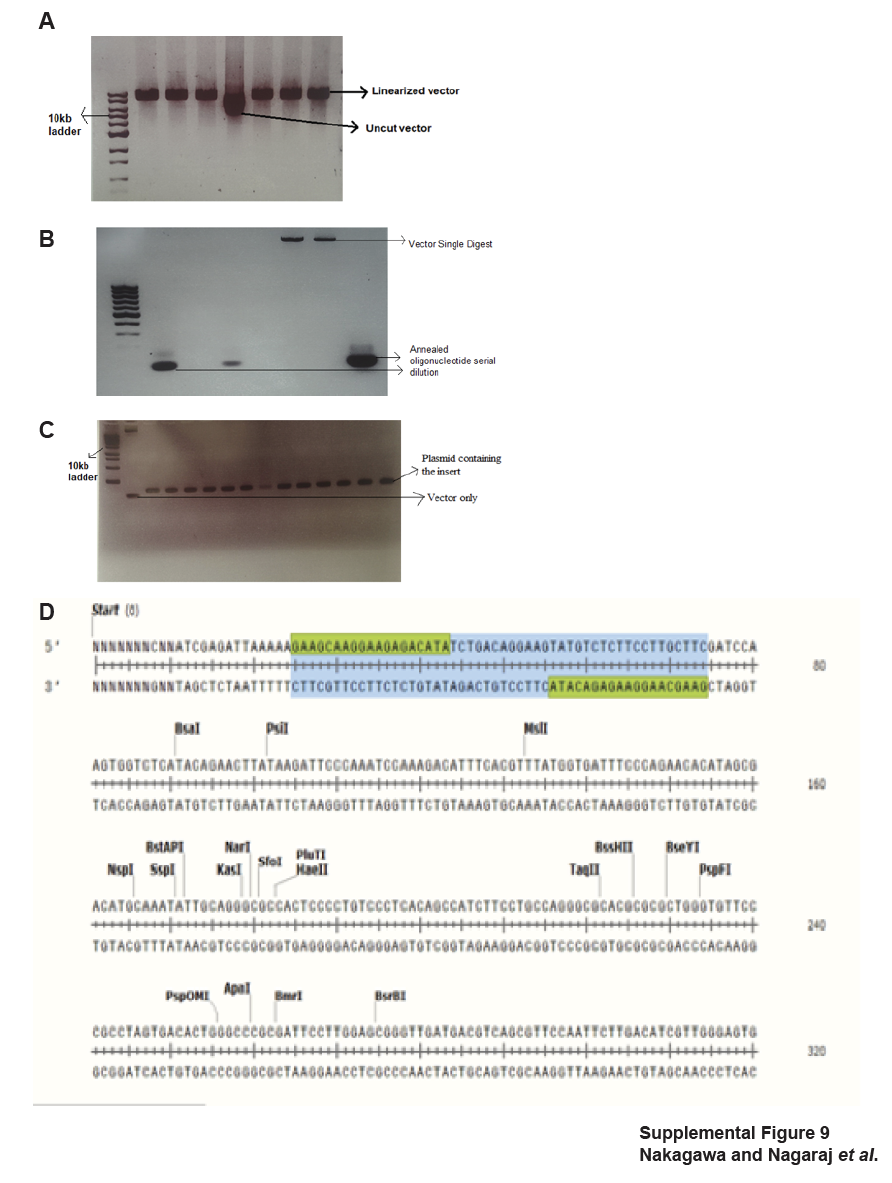
Suppl. Fig. 9. LncRNA β-CatM positively regulates EZH2-bound target genes.**

(**A**) Restriction digested vector which runs at a different rate when compared to the intact vector on an agarose gel.

(**B**) Annealed oligonucleotide ran on an agarose gel to check size and presence of oligonucleotide.

(**C**) Colony PCR which shows the presence of an insert in the test when compared to vector alone.

(**D**) Sequencing output which shows the presence of the target sequence which is highlighted in green.

**
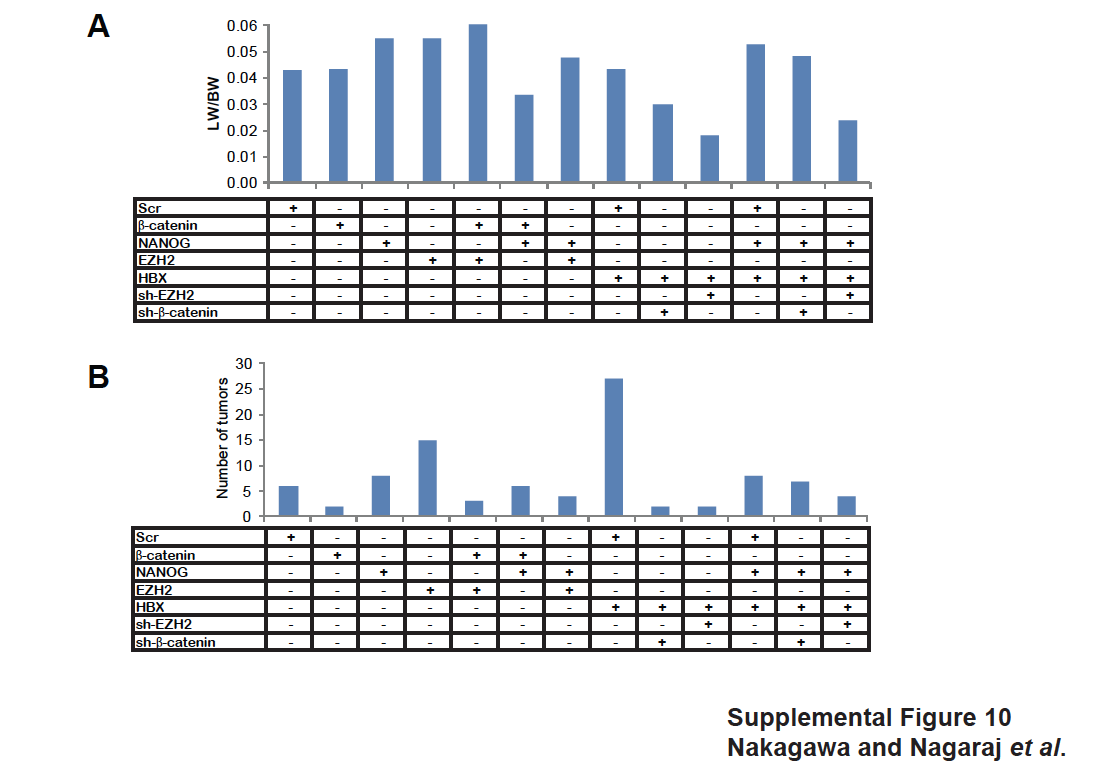
**

**Suppl. Fig. 10. HBx in the presence or absence of NANOG expression produced the highest tumor burden, while the other groups showed little to no tumor burden after intrasplenic injection into C57Bl/6 mice.**

(**A**) Liver weight to body weight ratio of mice after euthanasia at 3 months post intrasplenic injection. After two months, these animals were scanned for tumor growth using ultrasound and microCT.

(**B**) Liver images from euthanized mice (**left**) and number of tumor nodules found on each liver (right). The HBx + Scrambled shRNA (Scr) and HBx + NANOG + Scr groups had the highest tumor burden, while the other groups showed little to no tumor burden.

**Suppl. Table 1. KEY RESOURCES TABLE**

| **REAGENT or RESOURCE** | **SOURCE** | **IDENTIFIER** |
| --- | --- | --- |
| Immune Globulin Preparations | | |
| HBIG: 5% (50 mg/ml) | Biotest AG | Lot 130130 |
| Control IVIG (Bivigam): 10% (100 mg/ml) | Biotest AG | B584022 |
|  |  |  |
| **Antibodies** | | |
| Human *CD133* | Miltenyi or Becton Dickinson |  |
| Human *CD49f (Integrin α 6)* | Becton Dickinson |  |
| Human *CD45* | Becton Dickinson |  |
| NANOG | Abcam | ab80892 |
| HBs Ag | Pharmingen |  |
| Anti-human IgG APC | Miletini or Becton Dickinson |  |
| Anti-human IgG PE | Miletini or Becton Dickinson |  |
| β-Actin mouse Monoclonal Antibody | Santa Cruz | sc-69879 |
| Non-phospho (Active) β-Catenin (Ser33/37/Thr41) (D13A1) Rabbit mAb | Cell Signaling | 8814 |
| **Chemicals, and Recombinant Proteins** | | |
| Cycloheximide | Sigma Aldrich | 239764-100MG |
| Triton X-100 | Sigma Aldrich | T8787-50ML |
| Paraformaldehyde | Sigma Aldrich | P6148-500G |
| Polybrene | Sigma Aldrich | TR-1003 |
| Puromycin Dihydrochloride | Thermo Fisher | A1113803 |
| Protease Inhibitor | Roche | 4693159001 |
| Proteinase K | Roche | 3115879001 |
| One Shot™ Stbl3™ Chemically Competent E. coli | Thermo Fisher | C737303 |
| Stellar™ Competent Cells | Takara | 636766 |
| Q5® High-Fidelity DNA Polymerase | NEB | M0491L |
| T4 DNA Ligase | NEB | M0202L |
| Quick Blunting™ Kit | NEB | E1201L |
| GenClone Fetal Bovine Serum, Heat Inactivated | Genesee Scientific | 25-514H |
| Dulbecco’s modified Eagle’s medium (DMEM, High  Glucose, with L-Glutamine, with Sodium Pyruvate) | Genesee Scientific | 25-500 |
| Glutamax | Thermo Fisher | 35050061 |
| Antibiotic:Antimycotic Solution | Gemini | 400-101 |
| DPBS | VWR | VWRL0117-0500 |
| BioT | Bioland Scientific | B01-01 |
| 6X Laemmli Sample Buffer | Bioland Scientific | SAB03-01 |
| LB Broth (Miller) Mix | Genesee Scientific | 11-120 |
| LB Agar (Miller) Mix | Genesee Scientific | 11-122 |
| PVDF membrane | EMD Millipore | ISEQ85R |
| Nylon membrane | Thermo Fisher | AM10104 |
| X-ray film | Thermo Fisher | 34091 |
| TRIzol Reagent | Thermo Fisher | 15596026 |
| SuperScript III reverse transcriptase | Thermo Fisher | 18080085 |
| RNasin® Ribonuclease Inhibitors | Promega | N2511 |
| EvaGreen miRNA qPCR Master Mix | Genomics-online | ABIN4219203 |
| Brilliant II SYBR Green qPCR Master Mix | Stratagene | 600828 |
| SYBR Green PCR Master Mix | Thermo Fisher | 4309155 |
| Pierce ECL plus | Thermo Fisher | 32132 |
| Immobilon Western chemiluminescent HRP substrate | EMD Millipore | WPKLS0500 |
| Protein A/G PLUS Agarose | Santa Cruz | Sc-2003 |
|  |  |  |

| **Critical Commercial Assays** | | |
| --- | --- | --- |
| RIP-assay kit | MBL | RN1001 |
| RNeasy mini kit | QIAGEN | 74104 |
| QIAprep Spin Miniprep Kit | QIAGEN | 27106 |
| MinElute Gel Extraction Kit | QIAGEN | 28604 |
| Lenti-X™ GoStix | Takara | 631280 |
| Quickchange Lightning site-directed mutagenesis kit | Agilent | 210519 |
| Dual-Luciferase Reporter Assay System | Promega | E1960 |
| MAXIscript™ SP6/T7 Transcription Kit | Thermo Fisher | AM1322 |
| mirPremier microRNA Isolation Kit | Sigma Aldrich | SNC50-1KT |
| miRNA cDNA Synthesis Kit | ABM | G270 |
| Northern Blot Assay | Signosis | NB-3001 |
|  |  |  |
| **Deposited Data** | | |
|  |  |  |
| **Experimental Models: Cell Lines** | | |
| Human: HEK 293T | ATCC | ATCC |
| Human: Huh7 | Riken Bioresources | Riken Bioresources |
| Human: PH5CH | ATCC | ATCC |
|  |  |  |
| **Experimental Models: Organisms/Strains** | | |
| Mouse: *HBV Tg* | Gift from Dr. James Ou | N/A |
| Mouse: *HBV HBx-null Tg* | Gift from Dr. James Ou | N/A |
| Mouse: *NOD.cg-Prkdcscidil2rgtm1Wjl/SZJ* | Jackson Laboratory | 005557 |
|  |  |  |
|  |  |  |
|  |  |  |
| **Recombinant DNA** | | |
| pPAX2 | Addgene | Plasmid #12260 |
| pMD2.G | Addgene | Plasmid #12259 |
| scrambled shRNA | Addgene | Plasmid #1864 |
|  |  |  |
| **Software and Algorithms** | | |
| GraphPad Prism 6 | GraphPad Software | https://www.graphpad. com/scientific- software/prism/ |
| ImageJ | N/A | https://imagej.nih.gov/ij/ |
| MetaMorph | Molecular Devices LLC | MS-MM |
| ELDA | N/A | [http://bioinf.wehi.edu.a](http://bioinf.wehi.edu.a/) u/software/elda / |
| STATA | Stata corp LP College Station | Version 11.0 |

• **CONTACT FOR REAGENT AND RESOURCE SHARING**

Contact for Reagent and Resource Sharing

Further information and requests for resources and reagents should be directed to and will be fulfilled by the

Lead Contact, Keigo Machida (keigo.machida@med.usc.edu).

Supplemental Table 2. Primers.

| Gene | Primer |
| --- | --- |
| Human *Albumin* | F : 5’-CATGCCAAATTAGTGCAGGA-3’  R : 5’-GCTGGGGTTGTCATCTTTGT-3’[^3^](#_ENREF_3) |
| Human *Afp* (α-Fetoprotein*)* | F : 5’-GCAAAGCTGAAAATGCAGTTGA-3’  R : 5’-GGAAAGTTCGGGTCCCAAAA-3’[^4^](#_ENREF_4) |
| Human *Cd133* (*Prom1*) | F : 5’-TCATCGCTGTGGTCGTCATTG-3’  R : 5’-GTCCGCTGGTGTAGTGTTGTAG-3’[^5^](#_ENREF_5) |
| Human *Cytokeratin 19* (*Ck19, KRT19*) | F : 5’-TGCTGGATGAGCTGACTCTG-3’  R : 5-AATCCACCTCCACACTGACC-3’[^6^](#_ENREF_6) |
| Human *E-Cad* | F : 5’- GCAGAAGTGTCCCTGTTCCAG-3’  R : ATCATAGCTACAGACAATGGTTCTCCAGTTGCT-3’[^7^](#_ENREF_7) |
| Human *Gapdh* | F : 5’- ACCACAGTCCATGCCATCAC-3’  R : 5’- TCCACCACCCTGTTGCTGTA-3’[^3^](#_ENREF_3) |
| Human *NANOG* | F : 5'-TGAACCTCAGCTACAAACAG-3’  R : 5'-TGGTGGTAGGAAGAGTAAAG-3'[^8^](#_ENREF_8) |
| Human *Oct4* | F : : 5'-AGCGAACCAGTATCGAGAAC-3’  R : 5'-TTACAGAACCACACTCGGAC-3'[^8^](#_ENREF_8) |
| Human *Sox2* | F : 5'-AGCTACAGCATGATGCAGGGA-3'  R : 5'-GGTCATGGAGTTGTACTGCA-3'[^8^](#_ENREF_8)^,^ [^9^](#_ENREF_9) |

| **Suppl. Table 3: *In vivo* oncogenic analysis of HBV Tg mouse model** | | | | | | | |
| --- | --- | --- | --- | --- | --- | --- | --- |
| **DEN** | **Mouse group** | **HBV genes** | **# of mice with tumor**  [**^10^**](#_ENREF_10)**^,^** [**^11^**](#_ENREF_11) | **Tumor incidence**  [**^10^**](#_ENREF_10)**^,^** [**^11^**](#_ENREF_11) | **Tumor grading (0-3)** | **Pathology** | **HBV titer (copies/ml)**  [**^10^**](#_ENREF_10)**^,^** [**^11^**](#_ENREF_11) |
| - (8 months) | wtTg05 | wt | 1/9 | 11% | N.D. |  | ~10^9 |
| - (8 months) | wtTg08 | wt | 0/8 | 0% | N.D. |  | ~10^6 |
| DEN (8 months) | CD1/B6 | None | 1/32 | 3% | N.D. |  | 0 |
| DEN (8 months) | wtTg05 | wt | 10/11 | 91%* | 2.4 ± 0.7 | Oval cell expansion, BrdU(+) | ~10^9 |
| DEN (8 months) | wtTg08 | wt | 12/15 | 80%* | 2.2 ± 0.5 |  | ~10^6 |
| DEN (8 months) | mtTg04 | HBV(HBx-null) | 24/30 | 80% | 1.3 ± 0.3 |  | 3 X 10^7 |
| DEN (8 months) | mtTg31 | HBV(HBx-null) | 17/27 | 63%** | 1.2 ± 0.4* |  | 1 X 10^7 |
| DEN (8 months) | mtTg04/X | HBV(HBx-null) + HBx Tg | 16/18 | 89% | 2.4 ± 0.7 | Oval cell expansion | N.D. |
| DEN (8 months) | mtTg31/X | HBV(HBx-null) + HBx Tg | 12/12 | 100% | 2.5 ± 0.8 | Oval cell expansion | 4 X 10^7 |
| - (23-24 months) | Non-Tg | None | 5/33 | 15% | N.D. |  | 0 |
| - (23-24 months) | Tg04(S2) | HBV(Pre-S2 mutant) | 8/19 | 42% | 1.5 ± 0.3 | ER stress, CycD1(+) | 3 X 10^7 |
| - (23-24 months) | Tg07(S2) | HBV(Pre-S2 mut) | 22/30 | 73% | 1.5 ± 0.4 | ER stress, CycD1(+) | 9 X 10^6 |
| - (23-24 months) | wtTg05 | wt | 14/20 | 70% | 1.7 ± 0.5 | Oval cell expansion | ~10^9 |
| * P<0.00001: The P value was determined by comparison to the control CD1/B6 mouse group with DEN injection.  Note: Dr. Ben Yen’s Line4 preS2 mutant mouse line is different from Dr. Ou’s mtTg04 mouse line, which is HBx-null HBV Tg mouse.  ** P<0.02: The difference between mtTg31/X and mtTg31 was statistically significant. | | | | | | | |

###

| Supplemental Table 4. TICs isolated from HBV-associated human HCC specimens. | | | |
| --- | --- | --- | --- |
| Liver Tumor ID Number | HBV antigen positive | Affinity of HBIG binding to cells | NANOG expression after incubation compared to IVIG incubated cells |
| 1 | HBV | High | Reduced |
| 2 | HBV | High | Reduced |
| 3 | HBV | Low | Marginal |
| 4 | HBV | No | No effect |

Supplemental Table 5. Summary of experiments for FACS studies of bindings between TICs and HBIG for the MTT or spheroid assays.

| Treatment | Drug dose in cell culture | # of 6-well |
| --- | --- | --- |
| 1. Control | Vehicle (300 mM Glycine pH4.3) | 3 |
| 2. HBIG (Low) | 0.78 IU/ml (1500 IU in humans) | 3 |
| 3. HBIG (Middle) | 3.13 IU/ml (6000 IU in humans) | 3 |
| 4. HBIG (High) | 5.22 IU/ml (10,000 in humans IU) | 3 |
| 5. Control IVIG (Bivigam) | 12.5 mg/ml (400 mg/kg in humans) | 3 |
|  |  | Total 15 |

**Supplemental Table 6. Cytotoxicity of PRI-724 in a panel of seven HCC cell lines and two TICs.**

##
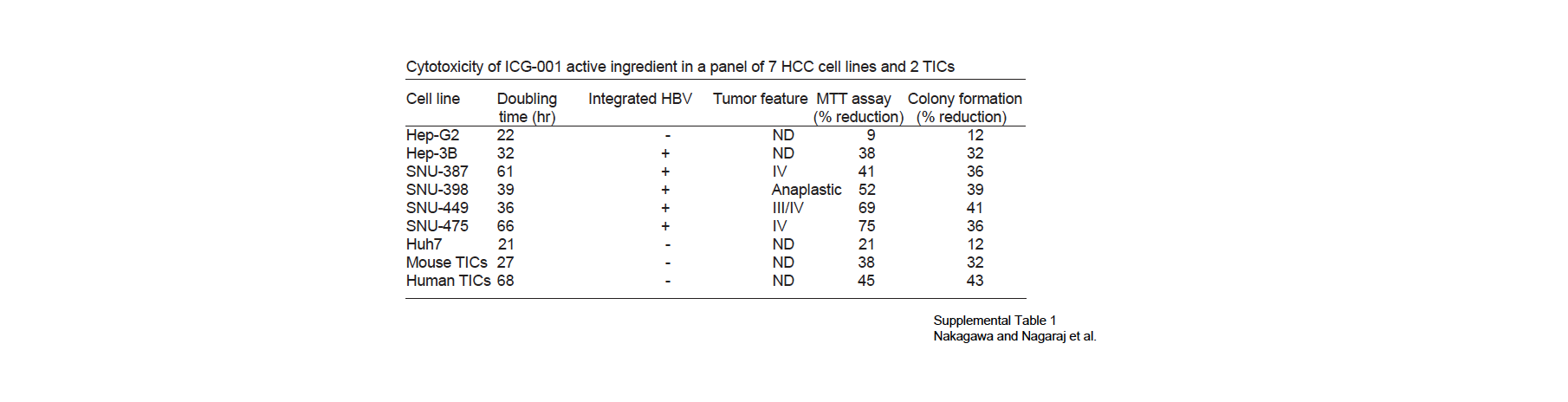


| **Suppl. Table 7. Breakdown of mice for therapy** | | | | | |
| --- | --- | --- | --- | --- | --- |
| **NOG Mouse Groups** | **Treatment** | **Binding affinity of TICs with HBIG** | **Viral antigen positivity** | **Endpoint**  **(month)** | **Total Mouse Number** |
| *1* | Vehicle | None | - | 3 | 5 mice |
| *2* | Vehicle | None | - | 3 | 5 mice |
| 7 | HBIG (High dose) | Low | - | 3 | 5 mice X 5 |
| 8 | HBIG (High dose) | Low | + | 3 | 5 mice X 5 |
| 9 | HBIG (High dose) | High | - | 3 | 5 mice X 5 |
| 10 | HBIG (High dose) | High | + | 3 | 5 mice X 5 |
| 11 | Control IVIG | None | - | 3 | 5 mice |
|  |  |  |  |  | 115 |

| **Suppl. Table 8. Summary of experiments for *in vivo* efficacy studies of HBIG in TICs engrafted in immunocompromised mice *(10 mice per dose level)*** | | |
| --- | --- | --- |
| Treatment | Drug dose (ip) |  |
| 1. Control | Vehicle |  |
| 2. HBIG (Low) | 1.56 IU/mouse: 5 μl (1500 IU in humans) |  |
| 3. HBIG (Middle) | 6.25 IU/mouse: 20 μl (6000 IU in humans) |  |
| 4. HBIG (High) | 10.4 IU/mouse: 33.3 μl (10,000 IU in humans) |  |
| 5. Control IVIG (Bivigam) | 25 mg/mouse (Equivalent to 400 mg/kg in humans) |  |
| Total mice |  |  |
| Peto’s Wilcoxon test for comparison of survival between groups. Probability values <0.05 was regarded as significant. | | |

| Suppl. Table 9. Proposed mechanisms for anti-tumorigenic effects of IVIG |
| --- |
| Direct antibody-mediated cytotoxicity |
| Complement dependent |
| Complement independent |
| Antibody-mediated cellular cytotoxicity |
| NK mediated |
| Activity against endothelial adhesion molecules |
| Activity against tumor adhesion machinery |
| Induction of cytokine secretion e.g. IL-12, transforming growth factor-β (enhancement of NK activity) |
| Anti-infective mechanisms (e.g. anti-HBV) |
| Masking of MHC-I and enhancing of NK cells anti-tumor activity |
| Anti-angiogenic effect |
| Miscellaneous |

**Supporting Materials and Methods**

**Reverse Transcription and Real-Time PCR (qPCR)**

Total RNA was extracted using TRIzol Reagent (Invitrogen) and purified using the RNeasy mini kit (QIAGEN) according to the manufacturer’s protocol. RNA concentration and purity were determined by A_260_ and A_260_/A_280_ ratios, respectively. The RNA samples were treated with DNase I (Invitrogen) to remove residual traces of DNA. cDNA was prepared from 1 µg of total RNA, using SuperScript III reverse transcriptase (Invitrogen) and random primers in a final volume of 10 µL. cDNAs were amplified by polymerase chain reaction (PCR) using the primer pairs listed below. Quantitative real-time PCR (qPCR) was performed on an ABI 7300 HT Real-Time PCR machine using 2X SYBR Green Master Mix (Applied Biosystems). Conditions for all reactions were as follows: 1 cycle at 50°C for 2 minutes, followed by 1 cycle at 95°C for 10 minutes, followed by 40 cycles at 95°C for 15 seconds and 60°C for 1 minute. Specificity of the PCR products was tested by thermal dissociation curves. Gene expression was determined as a relative ratio to glyceraldehyde 3-phosphate dehydrogenase control via the ΔC_t_ method. The data shown represent the means ± standard deviation (S.D.).

###

### **MTT Assay**

MTT assay was performed as previously described [^12^](#_ENREF_12)^,^ [^13^](#_ENREF_13). Briefly, the plate was inverted followed by a rapid flick to remove the medium plus any antibodies [^12^](#_ENREF_12). A total of 50 μL of 2 mg/mL MTT (Sigma Chemical Co. Ltd, Poole) in Hank's balanced salt solution without phenol red was added to each well, and the plate was reincubated at 37°C in 5% CO_2_ for a further 4 hours as previously described [^14^](#_ENREF_14). The formazan crystals formed were dissolved in 100 μL acid/alcohol (0.04 N HCl in isopropanol) or dimethyl sulfoxide for comparative experiments by mixing on a microshaker (Dynatech Labs, Ltd, Billingshurst) for 10 minutes. The plate was then read on a microplate reader at 570 nm. The number of live cells per well was calculated as a percentage of the control to measure cell survival after antibody exposure.

**Supplemental References**

1. Bedell VM, Wang Y, Campbell JM, et al. In vivo genome editing using a high-efficiency TALEN system. Nature 2012;491:114-8.

2. Stack RM, Lenschow DJ, Gray GS, et al. IL-4 treatment of small splenic B cells induces costimulatory molecules B7-1 and B7-2. J Immunol 1994;152:5723-33.

3. Tanimizu N, Saito H, Mostov K, et al. Long-term culture of hepatic progenitors derived from mouse Dlk+ hepatoblasts. J Cell Sci 2004;117:6425-34.

4. Rao MS, Khan AA, Parveen N, et al. Characterization of hepatic progenitors from human fetal liver during second trimester. World J Gastroenterol 2008;14:5730-7.

5. Snippert HJ, van Es JH, van den Born M, et al. Prominin-1/CD133 marks stem cells and early progenitors in mouse small intestine. Gastroenterology 2009;136:2187-2194 e1.

6. Rountree CB, Senadheera S, Mato JM, et al. Expansion of liver cancer stem cells during aging in methionine adenosyltransferase 1A-deficient mice. Hepatology 2008;47:1288-97.

7. Rosivatz E, Becker I, Specht K, et al. Differential expression of the epithelial-mesenchymal transition regulators snail, SIP1, and twist in gastric cancer. Am J Pathol 2002;161:1881-91.

8. Park IH, Zhao R, West JA, et al. Reprogramming of human somatic cells to pluripotency with defined factors. Nature 2008;451:141-6.

9. Chen S, Do JT, Zhang Q, et al. Self-renewal of embryonic stem cells by a small molecule. Proc Natl Acad Sci U S A 2006;103:17266-71.

10. Zheng Y, Chen WL, Louie SG, et al. Hepatitis B virus promotes hepatocarcinogenesis in transgenic mice. Hepatology 2007;45:16-21.

11. Na B, Huang Z, Wang Q, et al. Transgenic expression of entire hepatitis B virus in mice induces hepatocarcinogenesis independent of chronic liver injury. PLoS One 2011;6:e26240.

12. Denizot F, Lang R. Rapid colorimetric assay for cell growth and survival. Modifications to the tetrazolium dye procedure giving improved sensitivity and reliability. J Immunol Methods 1986;89:271-7.

13. Chen CL, Tsukamoto H, Liu JC, et al. Reciprocal regulation by TLR4 and TGF-beta in tumor-initiating stem-like cells. J Clin Invest 2013;123:2832-49.

14. Sargent JM, Taylor CG. Appraisal of the MTT assay as a rapid test of chemosensitivity in acute myeloid leukaemia. Br J Cancer 1989;60:206-10.
